# Protein, Calcium, Vitamin D Intake and 25(OH)D Status in Normal Weight, Overweight, and Obese Older Adults: A Systematic Review and Meta-Analysis

**DOI:** 10.3389/fnut.2021.718658

**Published:** 2021-09-10

**Authors:** Priya Dewansingh, Gerlof A. R. Reckman, Caspar F. Mijlius, Wim P. Krijnen, Cees P. van der Schans, Harriët Jager-Wittenaar, Ellen G. H. M. van den Heuvel

**Affiliations:** ^1^Research Group Healthy Ageing, Allied Health Care and Nursing, Hanze University of Applied Sciences, Groningen, Netherlands; ^2^Department of Internal Medicine, University of Groningen, University Medical Center Groningen, Groningen, Netherlands; ^3^Faculty of Mathematics and Natural Sciences, University of Groningen, Groningen, Netherlands; ^4^Faculty of Medical Sciences, University Medical Center Groningen, Groningen, Netherlands; ^5^Department of Rehabilitation Medicine, University of Groningen, University Medical Center Groningen, Groningen, Netherlands; ^6^Department of Health Psychology Research, University of Groningen, University Medical Center Groningen, Groningen, Netherlands; ^7^Department of Maxillofacial Surgery, University of Groningen, University Medical Center Groningen, Groningen, Netherlands; ^8^FrieslandCampina, Amersfoort, Netherlands

**Keywords:** overweight, obesity, protein, micronutrients, older adults

## Abstract

The aging process is often accompanied by increase in body weight. Older adults with overweight or obesity might have an overconsumption in energy that is accompanied by inadequate intake of protein, vitamin D, and calcium. It is unclear if intake of protein and vitamin D and calcium is sufficient in older adults with overweight/obesity, and whether it differs from older adults with normal weight, since a recent overview of the literature review is lacking. Therefore, we systematically analyzed the current evidence on differences in nutrient intake/status of protein, vitamin D and calcium between older adults with different body mass index (BMI) categories. Randomized controlled trials and prospective cohort studies were identified from PubMed and EMBASE. Studies reporting nutrient intake/status in older adults aged ≥50 years with overweight/obesity and studies comparing between overweight/obesity and normal weight were included. Nutrient intake/status baseline values were reviewed and when possible calculated for one BMI category (single-group meta-analysis), or compared between BMI categories (meta-analysis). Nutrient intake/status was compared with international recommendations. Mean protein (*N* = 8) and calcium intake (*N* = 5) was 0.98 gram/kilogram body weight/day (g/kg/d) [95% Confidence Interval (CI) 0.89–1.08] and 965 mg [95% CI: 704–1225] in overweight/obese. Vitamin D intake was insufficient in all BMI categories (*N* = 5). The pooled mean for vitamin D intake was 6 ug [95% CI 4–9]. For 25(OH)D, the pooled mean was 54 nmol/L [95% CI 45–62], 52 nmol/L [95% CI 46–58], and 48 nmol/l [95% CI 33–62] in normal (*N* = 7), combined overweight and obese (*N* = 12), and obese older adults (*N* = 4), respectively. In conclusion, older adults with overweight and obesity have a borderline sufficient protein and sufficient calcium intake, but insufficient vitamin D intake. The 25(OH)D concentration is deficient for the obese older adults.

## Introduction

In older adults with overweight or obesity, nutrient insufficiencies have been associated with a higher energy and fat intake, due to intake of food products with low nutrient density (1–3). The most frequently reported nutrient insufficiencies that have been associated with obesity are protein, vitamin D, and calcium. Insufficiencies of these nutrients, as well as overweight or obesity can have a negative effect on muscle strength (4, 5), physical function (5–7), and/or bone strength (8–10). To promote healthy aging by slowing down, halting, or reverting the process of deterioration in muscle strength, physical functioning, and bone strength in older adults, nutrient insufficiencies should therefore be prevented or treated.

Currently, an overview of the available studies on mean intake and serum values of protein, vitamin D, and calcium and the difference between older adults with overweight, obesity, and normal weight is lacking. To better guide healthcare professionals in effectively preventing and treating nutrient insufficiencies, various knowledge gaps need to be filled. First, consistent evidence is currently lacking on mean intake for protein, vitamin D, and calcium and 25-hydroxyvitamin D [25(OH)D] concentrations in older adults with overweight and obesity in comparison to their respective nutrient recommendations [e.g., from the European Society for Clinical Nutrition and Metabolism (11), the Institute of Medicine (12), and the European Food Safety Authority (13)]. Second, although other systematic reviews determined nutrient insufficiencies and deficiencies in the mainly obese population, it is currently unknown how large and severe the difference in nutrient intake and serum exactly is between older adults with overweight, obesity, and normal weight (14–18).

Therefore, in this systematic review combined with several meta-analyses, we aimed to compare the recent mean intake of protein, vitamin D, calcium, and 25(OH)D concentrations in older adults with overweight or obesity with nutrient recommendations, and to determine the difference in protein, vitamin D, calcium intake, and 25(OH)D concentrations between older adults with overweight, obese, and normal weight.

## Materials and Methods

### Protocol

This systematic review and meta-analysis was performed in accordance with the Preferred Reporting Items for Systematic Reviews and Meta-analyses (PRISMA) 2020 guidelines (19). The review (protocol) was not registered.

### Search Strategy

The electronic databases Embase and PubMed were searched, until 13 July 2021, using the following key words: Aged AND “Body Mass Index” OR overweight OR obese AND “vitamin D” OR calcium. A combination of medical subjecht headings (MeSH) terms and free text terms was used. The complete overview of the search strategy is presented in Supplementary File 1.

### Eligibility Criteria

Prospective cohort studies and randomized controlled trials (RCTs) were included if participants were older adults, i.e., aged ≥50 years, with overweight or obesity, or if studies included older adults with normal weight older adults, in addition to older adults with overweight or obesity. Only baseline data such as mean, standard deviation (SD) and sample size were extracted as the current systematic review and meta-analysis focused on habitual nutrient intake/status. Studies were excluded when participants had renal or kidney dysfunction. Studies from the last 10 years were included.

### Study Selection

The title and abstract of every retrieved study was independently screened for relevance and eligibility by two reviewers. After the title and abstract selection, the remaining full-text studies were screened. The reference lists from the included full-text studies were also screened on title, abstract, and full-text. In addition, reference lists of review studies were also screened for additional relevant studies based on title, abstract and full-text. Disagreement between the two reviewers was settled by discussion with the co-authors reaching consensus in all cases.

### Data-Extraction

After the screening process, study sample characteristics, i.e., gender, age, country, and outcome measurements were collected by the first author. Body mass index (BMI) was used to define normal weight, overweight, and obesity, according to the values 18.5–24.9, 25.0–29.9, and ≥30 kg/m^2^, respectively (20). Participants in the BMI category <18.5 kg/m^2^ were excluded, since they are generally considered as underweight (20). Studies that did not distinguish older adults between normal weight and underweight will be included. Outcomes for this study were: protein (g/day, g/kg body weight/day), vitamin D [microgram (μg), international units], calcium intake (milligram/day) and 25(OH)D concentrations in nanomole/liter (nmol/L). Missing or unclear information was mentioned in the evidence table.

### Quality Assessment

Quality assessment was performed by the first author. Since for RCTs the baseline data were of primary interest, the methodological quality was not tested for RCTs. Prospective cohort studies were assessed with the relevant part of Cochrane's Tool to Assess Risk of Bias in Cohort Studies (21). More specifically, the investigated questions were: (a) Was selection of exposed and non-exposed cohorts drawn from the same population? (b) Can we be confident in the assessment of exposure? (c) Did the study match exposed and unexposed for all variables that are associated with the outcome of interest or did the statistical analysis adjust for these prognostic variables? (d) Can we be confident in the assessment of the presence or absence of prognostic factors? (e) Was the follow up of cohorts adequate? [Higgins and Green (21)].

### Comparison of Nutrient Intake/Status With International Recommendations

To determine if the habitual nutrient intake or nutrient status was sufficient, intake/status was compared to recommendations from international guidelines. Table 1 shows the recommendations for protein, vitamin D, and calcium intake, and 25(OH)D concentration.

**Table 1:** The international recommendation for protein, vitamin D, and calcium intake, and 25(OH)D concentration.

**Nutrient intake/status**	**Recommendation**
Protein intake	1.0–1.2 g/kg/day (11)
Vitamin D intake	15–20 μg (22)
Calcium intake	950 mg (23)
25(OH)D	≥50 nmol/L ((22)

*25(OH)D, 25-Hydroxyvitamin D; g/kg, gram/kilogram body weight; μg, microgram; mg, milligram; nmol/L, nanomol/liter*.

### Statistical Analysis

To estimate pooled baseline means for the nutrient intake and nutrient status in the studies that included older adults who were overweight or obese, a single-group meta-analysis was performed using a random effects model (RE). Subgroups (e.g., gender-based) in studies were combined with respect to the mean, SD, and number of participants (n) according to formulas recommended by the Cochrane Handbook [Higgins and Green (21)].

In studies in which the older adults were distributed over multiple BMI categories, a meta-analysis was performed to investigate the mean difference, with corresponding 95% confidence interval (CI), between older adults with overweight, obesity, and normal weight. Meta-analysis was performed when at least three studies were available. Heterogeneity was identified byI^2^. I^2^ was interpreted according to the Cochrane handbook: 0 to 40% = might not be important; 30 to 60% = may represent moderate heterogeneity; 50 to 90% = may represent substantial heterogeneity; 75 to 100% = considerable heterogeneity (24).

Funnel plots were formed to identify possible publication bias (24). The single-group meta-analyses were performed in R version 3.4.0, and all other meta-analyses were performed in RevMan 5.3.

## Results

### Search Results

**Figure 1:**
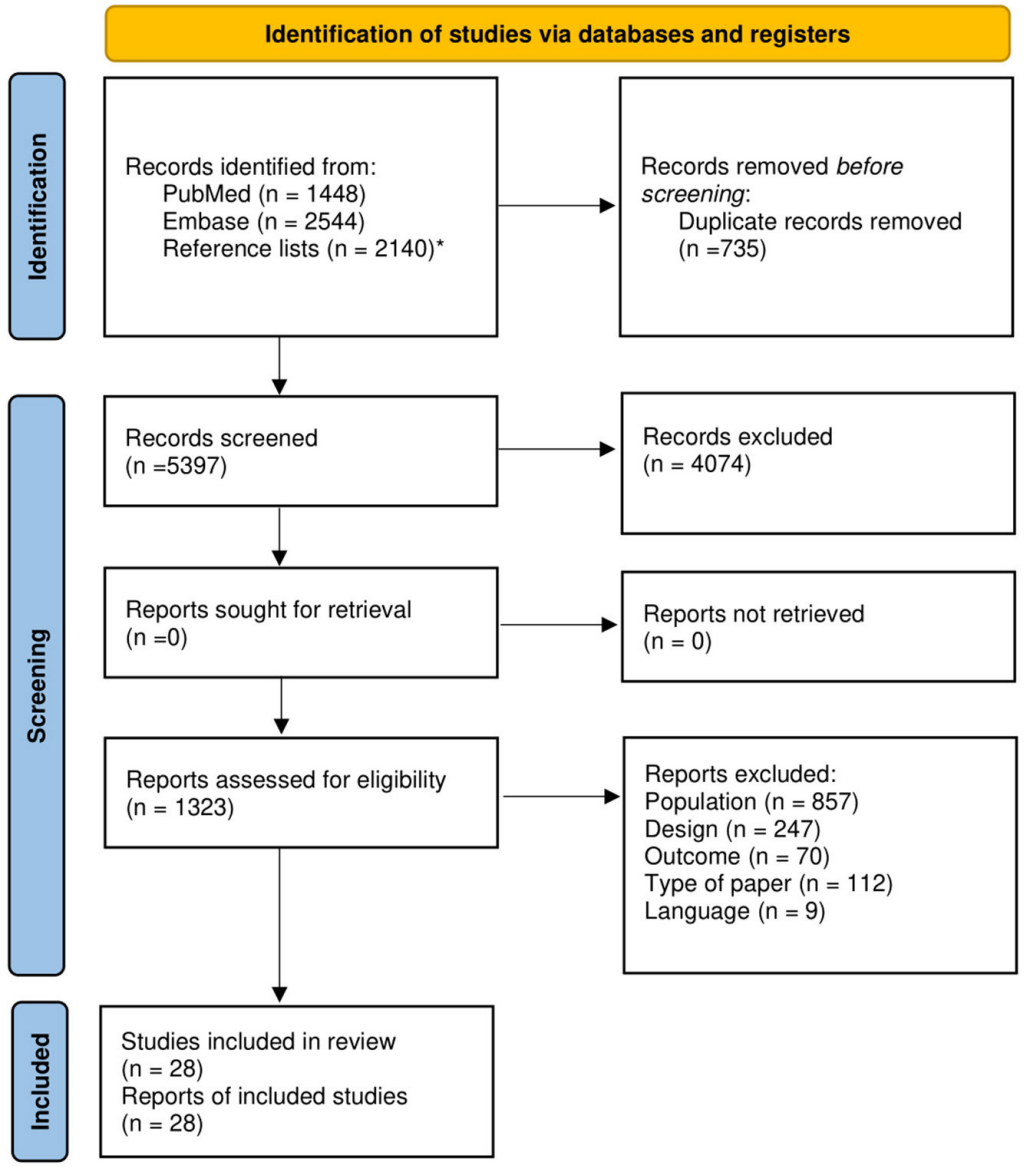
Flow-chart of study selection for systematic reviewing and meta-analysis. *The reference lists from the included full-text studies were also screened on title, abstract and full text.

The search in both databases indentified 3,992 studies, and the reference lists search identified 2,140 additional studies. From the total of 5,397 studies, 28 studies passed the inclusion criteria and were included in the systematic review of which 23 were suitable for meta-analyses, (Figure 1).

The 28 included studies comprised a total number of 45,814 older adults. Ten studies were found on intake of protein, five on vitamin D, seven on calcium intake, and 13 on 25(OH)D concentrations. Table 2 provides an overview of all studies. Single-group meta-analysis could be performed for the outcomes protein intake, calcium intake, and 25(OH)D concentrations, in older adults with overweight and obesity combined. For 25(OH)D concentrations, a separate single-group meta-analysis was performed comparing mean differences between older adults with obesity and normal weight.

**Table 2:** Overview of studies included in the systematic review and meta-analysis.

**References**	**Study population**	**Type of study**	**Outcome measurements**	**Conclusion**
Mason et al. (4)	218 postmenopausal overweight/obese women; 50–75 yrs old; United States of America	RCT	Vitamin D intake, vitamin D supplement intake, 25-Hydroxyvitamin D (25(OH)D), calcium intake	Dietary vitamin D intake and 25(OH)D was lower than the recommendation. Calcium intake was sufficient.
Sukumar et al. (10)	60 overweight/obese postmenopausal women; United States of America	Cohort	Vitamin D intake, 25(OH)D, calcium intake	Vitamin D intake was lower than the Daily recommended intake (DRI). Other nutrient intake and status were according to the DRI (25(OH)D and calcium intake).
Wood et al. (6)	305 healthy Scottish postmenopausal women (63% overweight/obese); 63.8 ± 2.2 yrs; Aberdeen, United Kingdom	RCT	25(OH)D, vitamin D intake, calcium intake	Vitamin D intake and 25(OH)D were lower than the recommendations in all Body mass Index (BMI) categories (normal weight, overweight, and obese). Calcium intake was sufficient in all BMI categories.
Sukumar et al. (25)	211 postmenopausal women (86% overweight/obese); BMI <25 kg/m^2^: 585 ± yrs; BMI 25–35 kg/m^2^: 585 ± yrs; BMI > 35 kg/m^2^:596 ± yrs; United States of America	RCT	Protein intake, calcium intake, vitamin D intake	25(OH)D and calcium intake was in favor of the BMI <25 kg/m^2^ category. For vitamin D intake, no differences between BMI categories were found.
Jesudason et al. (26)	323 overweight postmenopausal women; mean 59, standard error 0.4 yrs, BMI > 27 kg/m^2^; Australia	RCT	Protein intake, calcium intake, 25(OH)D	Protein intake, calcium intake and 25(OH)D was sufficient.
Rahme et al. (27)	257 overweight and obese elderly; 71.1 ± 4.8 yrs; Lebanon	RCT	Calcium intake, 25(OH)D	Calcium intake was lower than recommendation. 25(OH)D was sufficient.
Banitalebi et al. (28)	63 women with osteosarcopenic obesity, 64.1 ± 3.6 yrs; Iran	RCT	Calcium intake, vitamin D intake.	Calcium and vitamin D intake was lower compared to recommendations.
Verreijen et al. (29)	80 obese older adults; 63 ± 5.6 yrs; 53% female; the Netherlands	RCT	Protein intake	Protein intake was lower than the recommendation.
Dutheil et al. (30)	28 overweight/obese people, 19 males, 50–70 yrs; France	RCT	Protein intake	Protein intake was lower than the recommendation.
Leenders et al. (31)	60 overweight T2DM men; 71 ± 1 yrs, BMI 27.3 kg/m^2^ ± 0.4; the Netherlands	RCT	Protein intake	Protein intake was sufficient.
Backx et al. (32)	61 overweight and obese males (n = 36) and women (n = 25); between 55 and 70 yrs, BMI between 27and 40 kg/m^2^, waist circumference ≥102 cm for men, ≥88 cm for women; the Netherlands	RCT	Protein intake	Protein intake was sufficient.
Mojtahedi et al. (33)	31 overweight/obese postmenopausal women; mean: 65.2 ± 4.6 yrs; United States of America	RCT	Protein intake	Protein intake was lower than the recommendation.
Verreijen et al. (34)	100 overweight/obese adults (66% obese); 62.4 ± 5.4 yrs; the Netherlands	RCT	Protein intake	Protein intake was lower than the recommendation.
Smith et al. (35)	34 postmenopausal women with obesity; 50–65 yrs; United States of America.	RCT	Protein intake	Protein intake was lower than the recommendation.
Porter Starr et al. (36)	39 frail obese older adults; 68.3 ± 5.6 yrs; United States of America	RCT	Protein intake	Protein intake was lower than the recommendation.
Amamou et al. (37)	26 overweight sedentary men and women; 65.8 ± 3.1 yrs; Canada	RCT	Protein intake	Protein intake was sufficient.
Tremblay et al. (38)	100 men (44%) and women with metabolic syndrome, BMI: 33.4 ± 4.1 kg/m^2^; 59.4 ± 5.1 yrs; United Kingdom	RCT	Protein intake	Protein intake per day was lower than the recommendation.
Deibert et al. (39)	40 overweight males; 50–65 yrs; Germany	RCT	Protein intake	Protein intake was higher than the recommendation.
Dennison et al. (40)	913 overweight people, 465 males, 64.9 yrs; United Kingdom	Cohort	Calcium intake	Calcium intake was sufficient
Arabi et al. (41)	219 overweight and obese older adults, 43% men; 71.0 ± 4.7 yrs; Lebanon	RCT	25(OH)D	25(OH)D was lower than the recommendation.
Sohl et al. (5)	older adults from LASA 1 (*n* = 1,235, 66% overweight/obese), LASA 2 (*n* = 365, 71% overweight/obese) and B-PROOF study (182); 75.4 ± 6.5 yrs, 65.6 ± 2.9 yrs, 73.56 ± 3 yrs, respectively. The Netherlands	Cohort	25(OH)D	25(OH)D was sufficient.
Sohl et al. (9)	1,164 older Dutch people (66% overweight/obese); 75.2 ± 6.5 yrs; the Netherlands	Cohort	25(OH)D	older adults with BMI ≥ 25 kg/m^2^ had lower 25(OH)D levels than people with BMI <25 kg/m^2^.
Holecki et al. (42)	35 obese women (74%) and 19 non-obese women; obese: 53 yrs (range 52–55), non-obese: 54 yrs (range 51–56); Poland.	Cohort	25(OH)D3	25(OH)D was sufficient
Chlebowski et al. (7)	36,282 postmenopausal women (73% overweight/obese); 50–79 yrs; United States of America	RCT	25(OH)D	25(OH)D was sufficient in normal weight and overweight women. 25(OH)D was insufficient for postmenopausal women with obesity.
Scott et al. (43)	611 sarcopenic obese and non-sarcopenic obese older men; ≥70 yrs; Australia	Cohort	25(OH)D	25(OH)D was sufficient.
Haywood et al. (44)	117 obese community dwelling older adults (BMI ≥ 32 kg/m^2^); Australia	RCT	25(OH)D	25(OH)D was sufficient.
Pop et al. (45)	58 healthy overweight/obese women; 58 ± 6 yrs; United States of America	RCT	25(OH)D	25(OH)D was sufficient.
Olmos et al. (46)	2,597 men and postmenopausal women with normal weight: 61.0 ± 10.2 yrs, overweight: 64.9 ± 9.5, and obesity: 65.9 ± 9.5 yrs); Spain	Cohort	25(OH)D	25(OH)D was significantly lower in the obese compared to normal weight (*p* < 0.001) and overweight older adults (*p* < 0.001).

*Yrs, Years; 25(OH)D, 25-Hydroxyvitamin D; RCT, Randomized controlled trial; BMI, Body mass index; DRI, Daily recommended intake*.

### Risk of Bias

Seven cohort studies were assessed for risk of bias for population selection, assessment of exposure, match between exposed and unexposed for all variables, and presence or absence of prognostic factors (Table 3). All studies had a low risk of bias for population selection and assessment of exposure. For match between exposed and unexposed for all variables, and presence or absence of prognostic factors, two and five studies, respectively, scored a low risk of bias.

**Table 3:** Risk of bias per cohort study.

**References**	**Population selection^a^**	**Assessment of exposure^b^**	**Match exposed and unexposed for all variables^c^**	**Presence or absence of prognostic factors^d^**
Sohl et al. (5)	Yes	Definitely yes	Definitely no	Mostly yes
Holecki et al. (42)	Probably yes	Definitely yes	Mostly no	Definitely no
Dennison et al. (40)	Definitely yes	Probably yes	Not applicable	Not applicable
Sukumar et al. (10)	Probably yes	Definitely yes	Mostly yes	Mostly yes
Sohl et al. (9)	Definitely yes	Probably yes	Mostly no	Definitely no
Olmos et al. (46)	Probably yes	Definitely yes	Not applicable	Definitely yes
Scott et al. (43)	Probably yes	Definitely yes	Not applicable	Definitly yes

a *Was selection of exposed and non-exposed cohorts drawn from the same population?*

b *Can we be confident in the assessment of exposure?*

c *Did the study match exposed and unexposed for all variables that are associated with the outcome of interest or did the statistical analysis adjust for these prognostic variables?*

d *Can we be confident in the assessment of the presence or absence of prognostic factors?*

### Protein Intake

**Figure 2:**
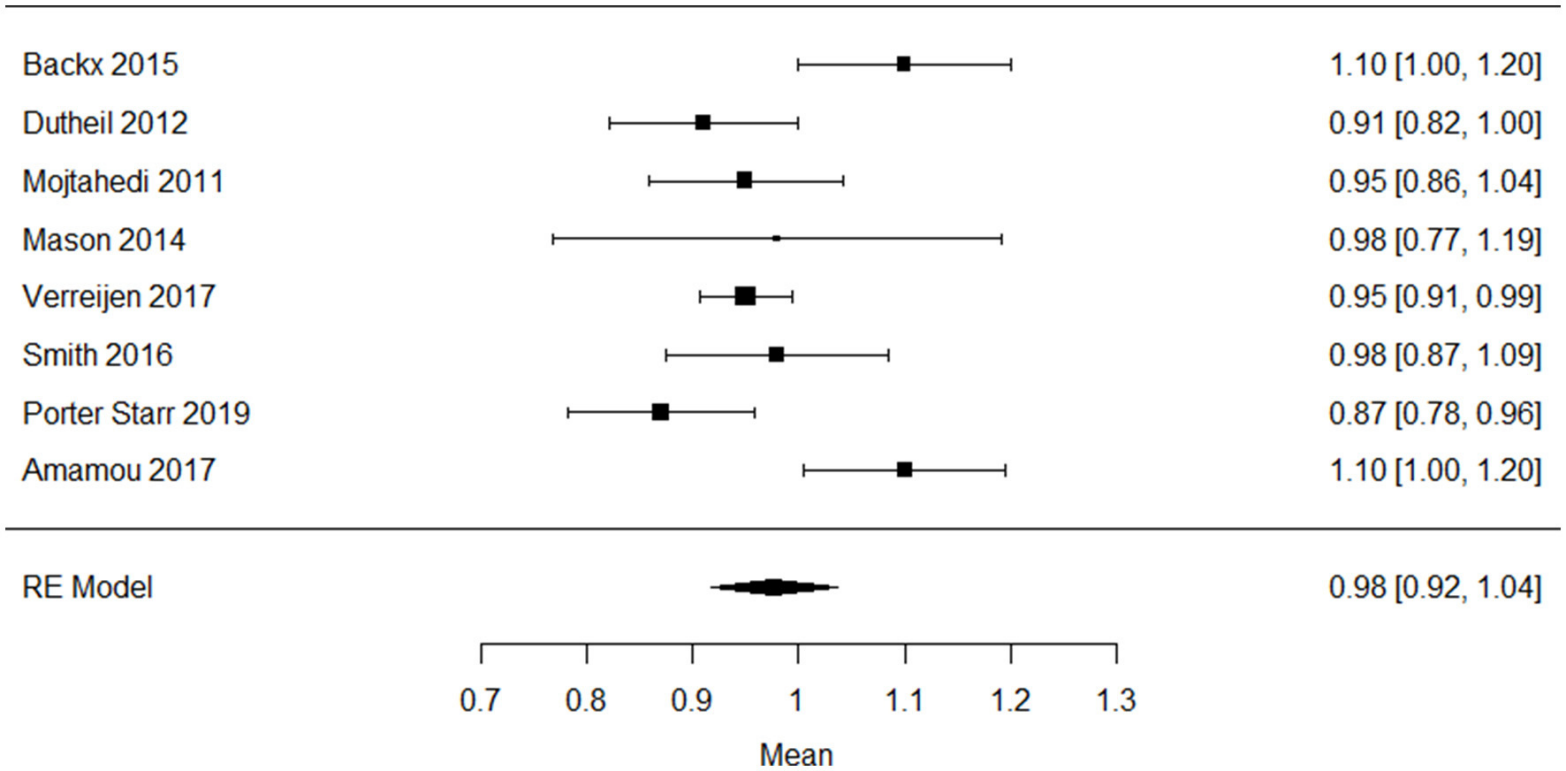
Forest plot of included studies on protein intake (g/kg/day) in older adults with overweight/obesity mean [95% confidence interval], RE, random effects.

All older adults (*n* = 988) in the 10 studies regarding protein intake were either overweight or obese. The mean protein intake (g/kg body weight/day) pooled from eight studies of 522 older adults with overweight/obesity was 0.98 g/kg body weight (g/kg)/day; 95% CI: 0.92–1.04), I^2^ = 71%, *p* = 0.0036 (Figure 2). The funnel plot (Supplementary Figure 2) on protein intake in older adults with overweigh/obesity showed a small asymmetry.

**Figure 3:**
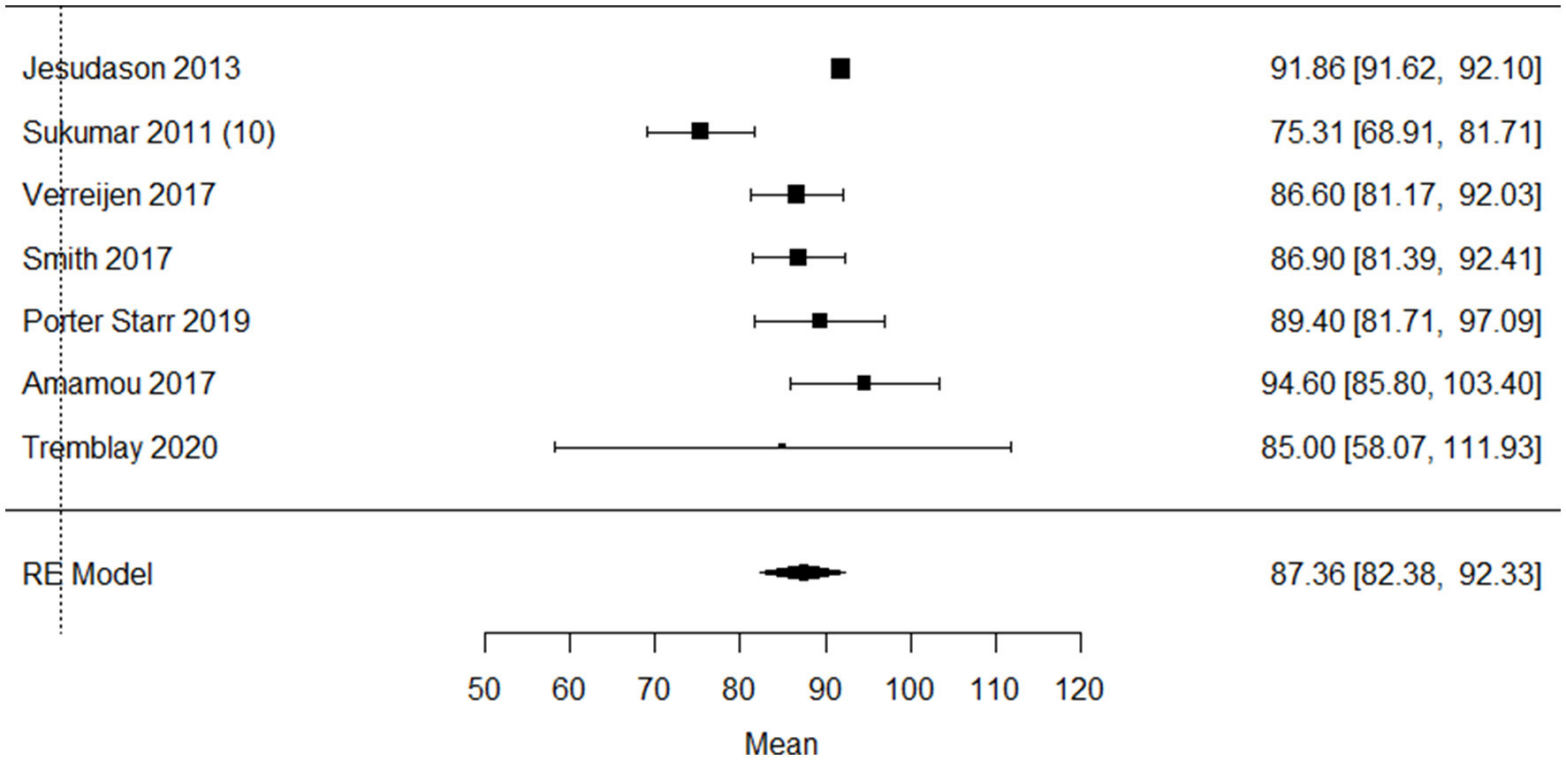
Forest plot of included studies on protein intake (g/day) in older adults with overweight/obesity mean [95% confidence interval], RE, random effects.

Seven studies reported protein intake as g/day in older adults with overweight and obesity, with a pooled mean intake of 87.4 mg; 82.4–92.3), I^2^ = 83% g/day, *p* < 0.0001 (Figure 3) (10, 26, 34–36, 38, 47). The funnel plot (Supplementary Figure 3) on protein intake (g/day) in older adults with overweigh/obesity showed a small asymmetry.

### Vitamin D Intake and 25(OH)D Concentrations

**Figure 4:**
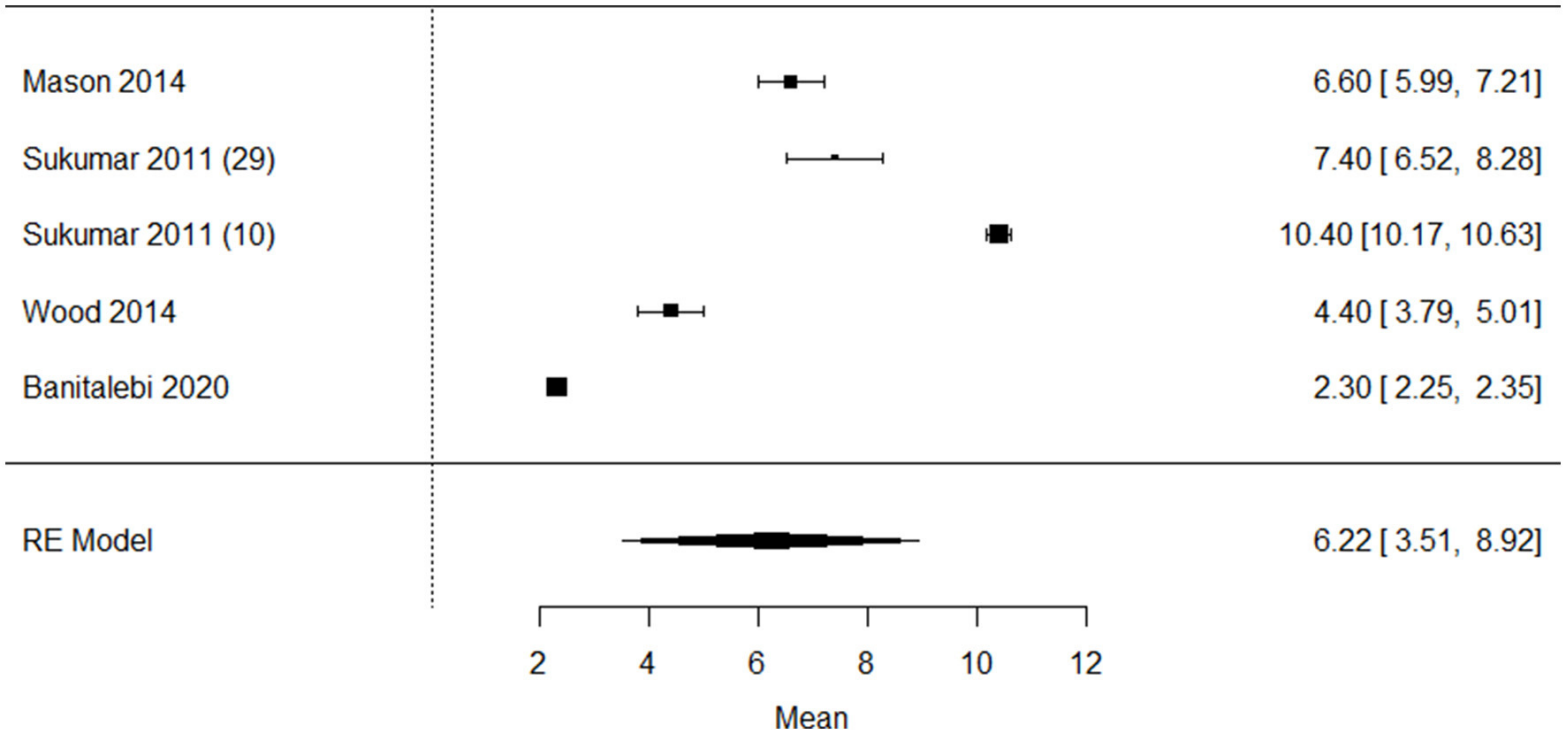
Forest plot of included studies on vitamin D intake (ug/day) in older adults with overweight/obesity mean [95% confidence interval], RE, random effects.

Five studies (*n* = 632) reported the baseline vitamin D intake. The pooled single-group meta-analysis in Figure 4 showed a mean vitamin D intake of 6.2 μg (95% CI: 3.51–8.92), I^2^ = 99.8%, *p* < 0.001 in older adults with overweight/obesity (4, 6, 10, 25, 28). The funnel plot (Supplementary Figure 4) on vitamin D intake in older adults with overweight/obesity showed asymmetry, indicating possible weak publication bias. Two studies investigated the difference in vitamin D intake between BMI categories. Neither of these studies reported significant differences between the older adults with overweight, or obesity and normal weight (6, 25).

**Figure 5:**
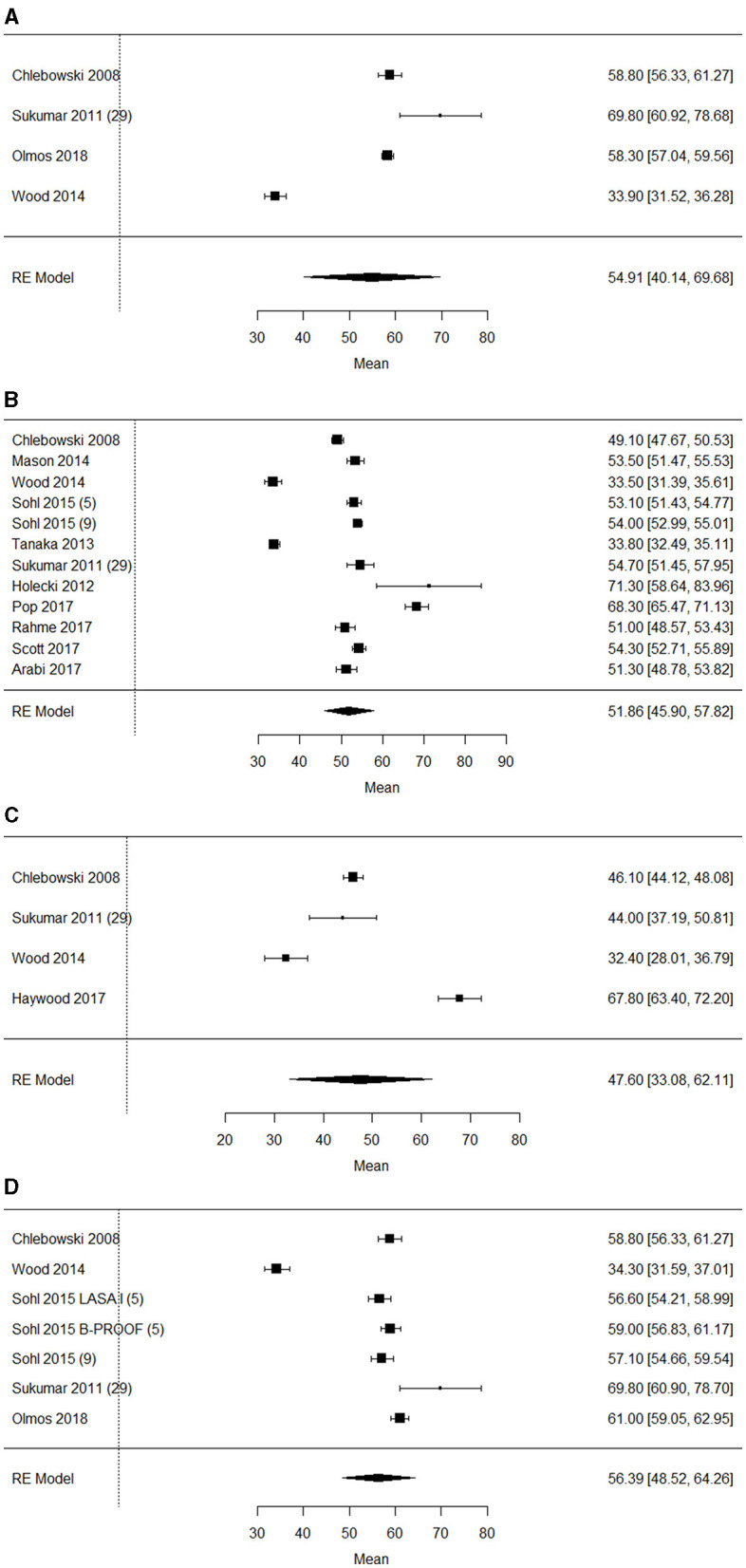
**(A)** Forest plot of included studies on 25(OH)D concentrations (nmol/L) in older adults with overweight mean [95% confidence interval], RE, random effects. **(B)** Forest plot of included studies on 25(OH)D concentrations (nmol/L) in older adults with overweight/obesity mean [95% confidence interval], RE, random effects. **(C)** Forest plot of included studies on 25(OH)D concentrations (nmol/L) in older adults with obesity mean [95% confidence interval], RE, random effects. **(D)** Forest plot of included studies on 25(OH)D concentrations (nmol/L) in older adults with normal weight mean [95% confidence interval], RE, random effects.

Fourteen studies that reported baseline 25(OH)D concentrations were included (4–9, 25, 41–45, 48). Nine of these compared older adults with overweight or obesity with older adults with normal weight. Four studies showed a significantly lower mean vitamin D status in older adults with overweight and obesity when comparing with normal weight older adults (6, 7, 25, 46). However, one study in overweight and obese Caucasian postmenopausal Scottish women (6) reported the lowest 25(OH)D concentrations (32.4 nmol/L, respectively), and indicated no significant differences in vitamin D status between older women with overweight, obesity and normal weight. 25(OH)D concentrations were obtained between January and March (6).

pooled single-group meta-analysis in Figure 5 showed a mean 25(OH)D concentration of 54.9 nmol/L (95% CI: 40.1–69.7), I^2^ = 99.3%, *p* < 0.001 in 1,639 older adults with overweight (Figure 5A), 51.9 nmol/L (95% CI: 45.9–57.8), I^2^ = 98.9%, *p* < 0.001 in 5,417 older adults with overweight/obesity (Figure 5B), 47.6 nmol/L (95%CI 33.1–62.1), I^2^ = 98.2%, *p* < 0.001) in 583 older adults with obesity (Figure 5C), and 53.6 nmol/L (95% CI: 44.8–62.4), I^2^ = 99.4%, *p* < 0.001 in 3,446 older adults with normal weight (Figure 5D), respectively. The funnel plot on 25(OH)D concentrations in older adults with overweight (Supplementary Figure 5), overweight/obesity (Supplementary Figure 6), obesity (Supplementary Figure 7), and normal weight (Supplementary Figure 8) showed asymmetry, indicating possible publication bias.

**Figure 6:**
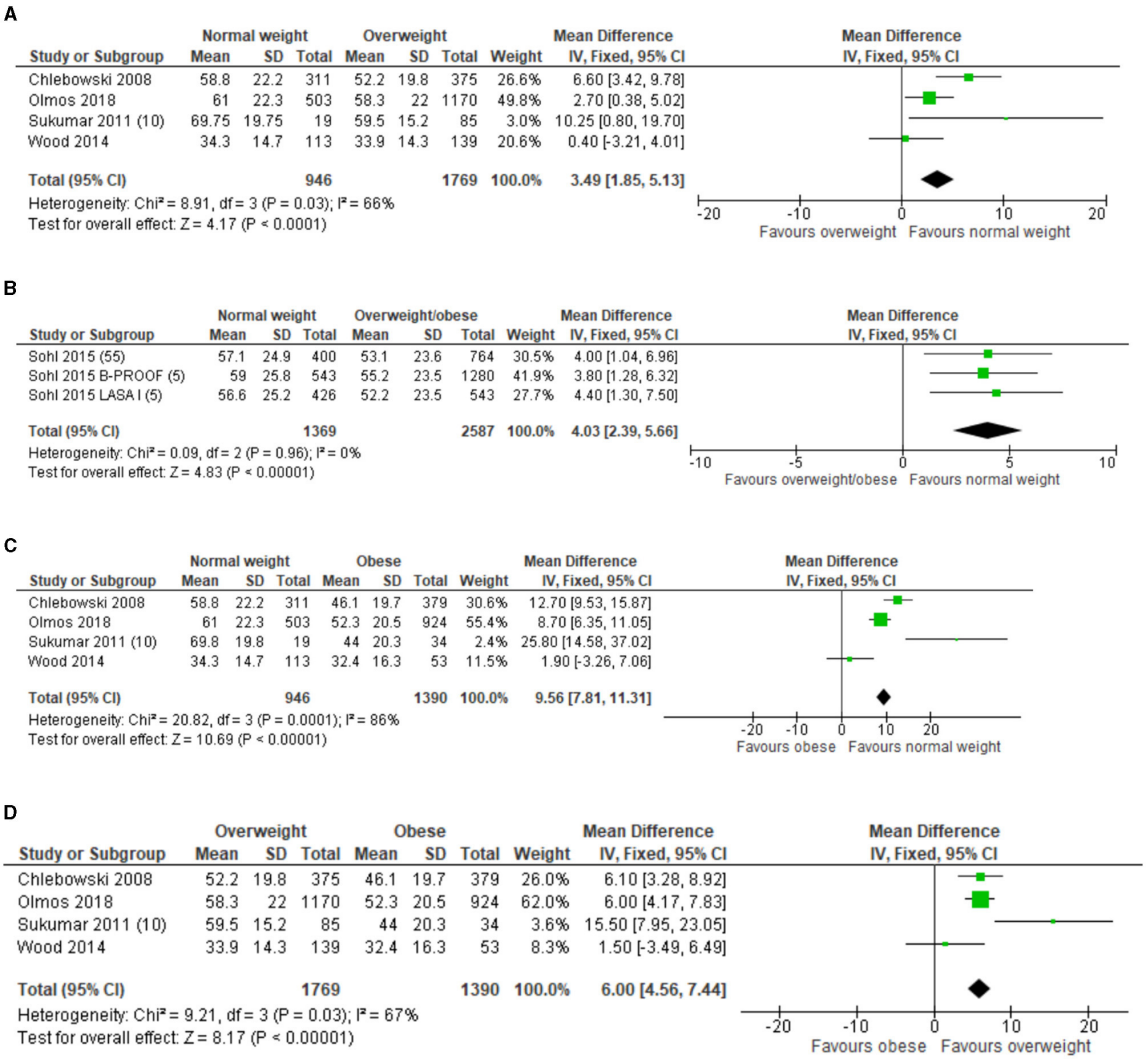
**(A)** Forest plot of included studies on 25(OH)D concentrations (nmol/L) in older adults between BMI categories overweight compared to normal weight. SD, standard deviation; IV, inverse varience; CI, confidence interval. **(B)** Forest plot of included studies on 25(OH)D concentrations (nmol/L) in older adults between BMI categories overweight/obese compared to normal weight. SD, standard deviation; IV, inverse varience; CI, confidence interval. **(C)** Forest plot of included studies on 25(OH)D concentrations (nmol/L) in older adults with between BMI categories obesity compared to normal weight. SD, standard deviation; IV, inverse varience; CI, confidence interval. **(D)** Forest plot of included studies on 25(OH)D concentrations (nmol/L) in older adults with between BMI categories overweight compared to obesity. SD, standard deviation; IV, inverse varience; CI, confidence interval.

Figure 6 shows results of the meta-analyses for 25(OH)D concentrations between older adults with overweight (Figure 6A), overweight/obese (Figure 6B), obese (Figure 6C) and normal weight (Figure 6D). The meta-analysis on baseline 25(OH)D concentrations showed that in three studies (*n* = 3,956), the older adults with overweight/obesity had lower 25(OH)D concentrations than the older adults with normal weight. The largest difference was found between older adults with obesity and normal weight (*n* = 899) with a MD of 9.56 nmol/L; 95% CI: 7.81–11.31, I^2^ = 86%, p <0.001. The funnel plot (Supplementary Figures 9–12) on 25(OH)D concentrations between BMI categories showed a small asymmetry, indicating possible weak publication bias.

### Calcium Intake

**Figure 7:**
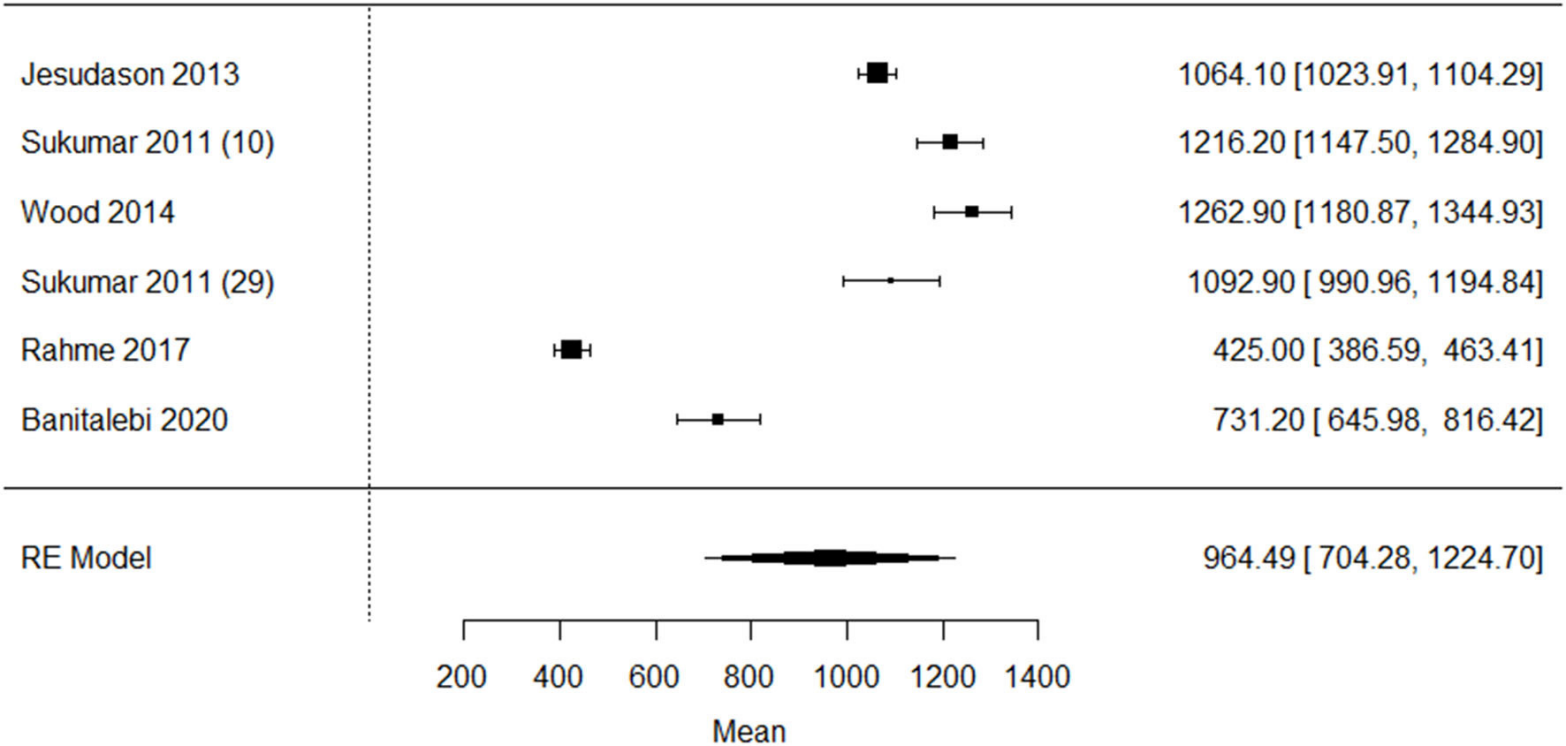
Forest plot of included studies on calcium intake (milligram/day) in older adults with overweight/obesity mean [95% confidence interval]. RE, Random effects.

In total, seven studies (*n* = 1,869) reported baseline intake for calcium. Five studies included only older adults who were overweight (40), obese (26, 28), or both (10, 27). Two studies included older adults with overweight, obesity, and normal weight, and ascertained a lower calcium intake in postmenopausal women with a BMI >35 kg/m^2^ compared to postmenopausal women with a BMI <25 kg/m^2^ (*p* < 0.02) (25). The other study in postmenopausal women reported no significant differences between older adults with overweight, obesity, and normal weight (6). Single-group meta-analysis in older adults with overweight and obesity was performed in six studies and showed a pooled mean calcium intake of 964.5.0 mg (95% CI: 704.3–1224.7), I^2^ = 99.1%, *p* < 0.001 (Figure 7). The funnel plot (Supplementary Figure 13) on calcium intake in older adults with overweight/obesity showed asymmetry, indicating possible publication bias.

**Figure 8:**
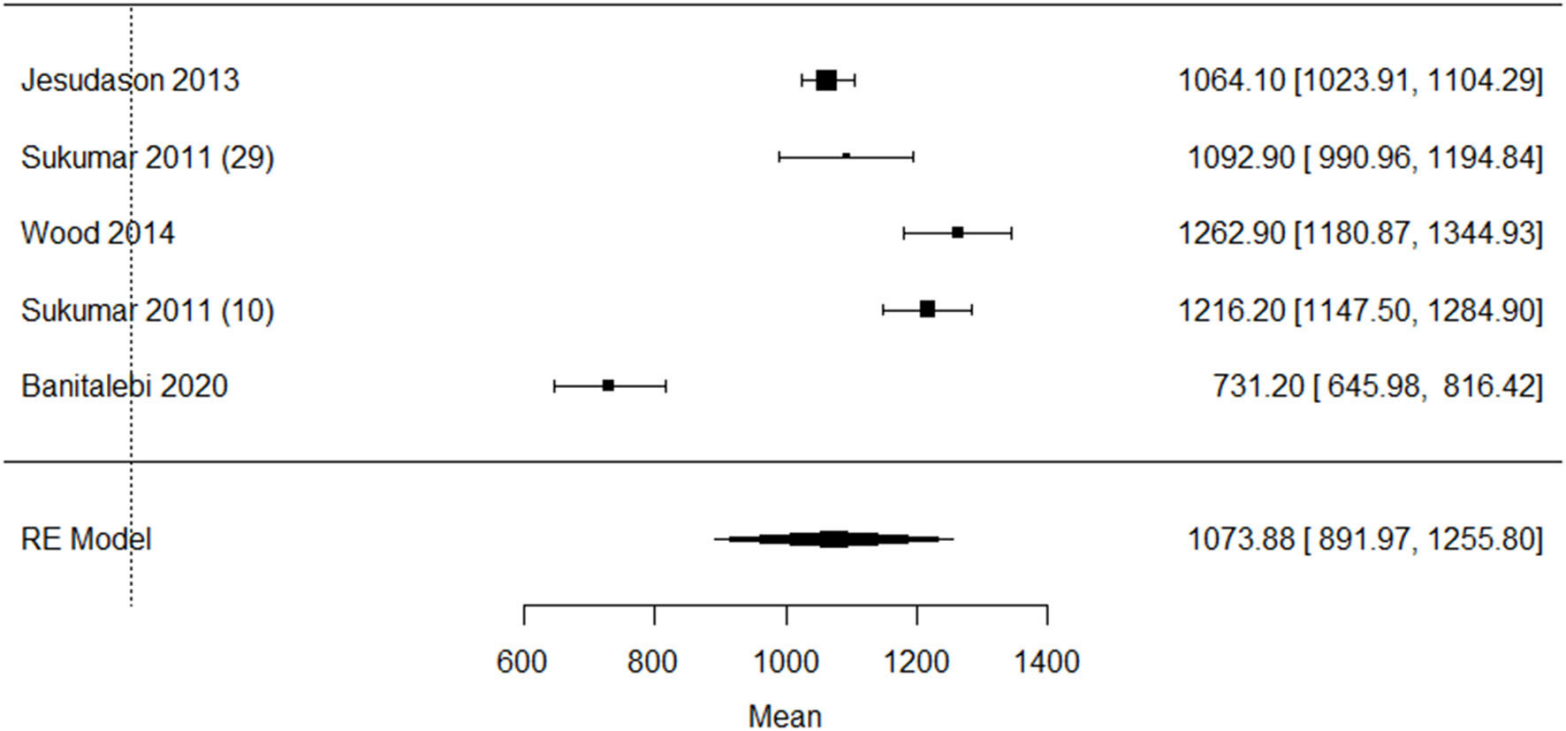
Sensitivity analysis of included studies on calcium intake (milligram/day) in older adults with overweight/obesity mean [95% confidence interval]. RE, Random effects.

One study in Lebanese older adults reported the very low mean dietary calcium intake (425 ± 292 mg) (27) compared to the other studies. Since it is known that the Lebanese population has a low calcium intake due to lower intake of dairy (49), this study was excluded from the meta-analysis (Figure 8). The sensitivity analysis showed a asymmetry, indicating possible publication bias (Supplementary Figure 14).

## Discussion

This is the first systematic review combined with a meta-analysis that compared the mean intake of protein, vitamin D, calcium, and 25(OH)D concentrations in older adults with overweight or obesity with nutrient recommendations, and determined the difference in protein, vitamin D, calcium intake, and 25(OH)D concentrations between older adults overweight, obesity, and normal weight. We found that the pooled mean protein intake is borderline sufficient when compared to the recommendations for a general healthy population. No studies were found with a focus on differences in protein intake between older adults with overweight, obesity, and normal weight. Vitamin D intake is insufficient in older adults across all BMI categories. Only in older adults with obesity, the pooled mean 25(OH)D concentration is deficient. Unfortunately, a meta-analysis could not be performed on the differences in protein, vitamin D, and calcium intake between older adults with overweight, obesity, and normal weight, due to the small number of studies available.

This study showed that the pooled mean of protein intake in older adults with overweight or obesity is almost equal to the recommendation from the European Society for Clinical Nutrition and Metabolism (ESPEN) of 1.0–1.2 gram protein/g/kg/day for healthy older adults (11). However, since the average pooled protein intake is bordeline sufficient, this strongly suggests that still a large proportion of these populations does not consume a sufficient daily amount of protein. The ESPEN recommendation for protein intake differs from the general EFSA recommendation for healthy adults, which is 0.83 g/kg/day (13). The ESPEN recommendation was based on the possible development of anabolic resistance in older adults (50, 51). The latter may result from underlying mechanisms such as increased splanchnic sequestration of amino acids, decreased postprandial availability of amino acids, lower postprandial perfusion of muscle, decreased muscle uptake of dietary amino acids, reduced anabolic signaling for protein synthesis, and reduced digestive capacity (50, 52, 53). Therefore, older adults are likely to benefit from a higher amount of protein intake per day. Data from the National Health and Nutrition Survey (NHANES) showed that 10–25% of the older adults in all BMI categories have a protein intake lower than the recommended dietary allowance of 0.8 g/kg/day (13, 54). This percentage seems lower compared to our results. The low prevalence of deficient protein intake in older adults from the NHANES study could be explained by the use of a different protein recommendation per day. In our study, we compared the pooled mean protein intake with the recommendation of 1.0 g protein/kg/day, while the NHANES compared the protein intake of their participants with the protein recommendation of 0.8 g/kg/day.

This systematic review found that vitamin D intake is insuffient in older adults with overweight, obesity, and normal weight, while the pooled mean for calcium intake was sufficient in older adults with overweight and obese. One of the included studies in a Lebanese elderly population showed a mean dietary calcium intake of 425 mg per day, indicating a very insuffient mean intake of dietary calcium in Lebanese elderly when compared to the EFSA recommendation for daily calcium intake of 950 mg (23).

Another study in Lebanese adults also demonstrated low dietary calcium intake in Lebanese adults, which was explained by a low intake of animal calcium sources, i.e., dairy (49). In the current study, mean vitamin D intake was insufficient in all weight groups, while the pooled mean 25(OH)D concentrations in the overweight/obese combined was barely sufficient, and the pooled mean for older adults with obesity was deficient compared to the 25(OH)D recommendation by the Institute of Medicine of ≥50 nmol/L (12). The results of the current study on vitamin D and calcium intake are in agreement with those found in another study in Americans aged ≥19 years, which reported also insufficient vitamin D intake and supplement intake and sufficient calcium intake/supplement intake across all BMI categories (55). Another study also showed that obese adults have a lower intake of dietary supplements compared to overweight, and normal weight adults (56–58). In the current study, older adults with obesity also had the highest deficiencies. Therefore, healthcare professionals may broaden their attention to especially adults with obesity who are at higher risk of having insufficient intake of vitamin D compared to older adults with normal weight.

The current meta-analysis showed that the pooled mean for 25(OH)D concentrations was bordeline sufficient for older adults with overweight and normal weight and insufficient for older adults with obesity,. One study showed a very low mean 25(OH)D concentration of 33 nmol/L when compared to the other included studies and the recommendations of ≥50 nmol/L. This study was performed in healthy Scottish postmenopausal women and their 25(OH)D concentrations were measured in the winter (January–March). Although the study in Scottish postmenopausal women showed low 25(OH)D concentrations, the mean concentration was comparable with another study in Scottish women aged between 60 and 70 years old, namely 30 nmol/L. Possible explanations were given for low 25(OH)D concentrations in Scotland and include the very low yearly quota of sun and therefore solar radiation. Also due to the high latitude, a large additional loss of ultraviolet light (UVB) is seen in Scotland (59–61). Lastly, dietary intake of vitamin D and/or supplements are insufficient. Three studies showed a 25(OH)D concentrations range between 68 and 71 nmol/L (42, 44, 45), which was well above the pooled mean of 48–55 nmol/L for the older adults with obesity and overweight, respectively. The possible reasons for the higher mean 25(OH)D concentrations in these studies might be due to several reasons, like more sun exposure in countries like Australia (44), fortification of food products with vitamin D, e.g., milk in the USA (45), and/or vitamin D supplementation use (42, 44, 45). However, information on these factors is hardly discussed in the studies, which make forming conclusions difficult.

When evaluating vitamin D status, body weight and body composition, in particular adipose tissue, should be taken into account. Firstly, it is known that the overweight and obese engage less in (outside) physical activity (62), and are therefore less exposed to solar ultraviolet radiation, which lowers the cutaneous vitamin D3 synthesis (63). Accumulation of visceral adipose tissue and physical inactivity has shown to be associated (64, 65). Secondly, adipose tissue stores the fat-soluble vitamin D, which can result in lower 25(OH)D concentrations. Studies found that increased amounts of vitamin D have been found in adipose tissue while 25(OH)D concentrations were considered insufficient in individuals with obesity (66, 67).

Besides insufficient intake of nutrient dense products as a cause of nutrient insufficiencies, overweight and obese individuals are reported to have altered absorption, distribution, metabolism, excretion of micronutrients, or a combination of these (14). In contrast to insufficient intake, excessive intake of calories can lead to incomplete biochemical reactions that can produce toxic by-products. These by-products are hypothesized to create a vicious circle of more weight gain and the negative features that accompanies the obese population, such as depression, fatigue, and the metabolic syndrome (68). In addition, concentrations of different antioxidants, vitamins, and minerals are correlated with serum leptin concentrations, which is important for the regulation of food intake and energy expenditure. Changes in leptin concentrations can lead to changes in adipose tissue mass and can therefore trigger an (systemic) inflammatory response, which is a risk factor for obesity (15).

Previous research has shown that persons with a higher BMI may misreport dietary intake. Underreporting of energy (69) and protein intake has been associated with a higher BMI in adults. Several studies reported underestimation of protein intake by ~12–20% (70–72), which indicates that the results for protein intake from this meta-analysis might be an underestimation. Underestimation of the protein intake could lead to less older adults with overweight/obesity with insuffient protein intake. However, it is less clear if older adults with overweight or obesity also underreport calcium intake and vitamin D intake. One cross-sectional Canadian study in children and adults from all BMI categories (*n* = 16,190) showed underestimation of vitamin D and calcium intake by 7 and 8%, respectively (22). More research on the possibility of misreportinging micronutrients in adults with overweight, obesity, and normal weight is needed to correctly interpret habitual micronutrient intake.

The current systematic review has various implications for future research. First, we could only compare mean pooled data with reference values for nutrient intake and nutrient status. These reference values are primarily based on a healthy older population, as no reference values for older adults with overweight, obesity, or both are available. Specific reference values for with overweight and obesity can be nesscary, due to their reported altered absorption, distribution, metabolism, excretion of micronutrients, or a combination of these (14). Second, only a small number of studies determined the nutrient intake separately for with normal weight, overweight, and obesity. Therefore, additional studies are needed to allow for a more representative meta-analysis. Third, the heterogenity of the studies (I^2^) was considerable, i.e., 71–99%. This finding was not suprising since the study populations differed considerably by including healthy older adults, postmenopausal women, and different countries. The results of the meta analysis should therefore be interpretated with caution, for example for postmenopausal women.

The results of our systematic review and meta-analysis also have implications for daily practice. Awareness on older adults with overweight and obesity having have risk for nutrient defiencies even though their food intake might be higher than older adults with normal weight should be increased. Based on the current study, special consideration for deficiencies in protein, calcium, and vitamin D intake is needed to prevent decrease in healthy aging by deterioration in muscle strength, physical performance, and bone strength (4, 5, 7, 8). Also, older adults with overweight and obese should include food products high in protein (e.g., meat, fish, dairy, cheese, nuts, lentils, beans, and legumes) and calcium (e.g., dairy, cheese, vegetables, nuts, and legumes) in their diet. To increase vitamin D intake, vitamin D supplementation is recommended in most countries, next to sufficient sun exposure.

## Conclusion

In the current systematic review combined with meta-analysis, we demonstrated that on average, older adults with overweight and obesity have a bordeline suffient protein intake, insufficient vitamin D intake and sufficient calcium intake. Although the average intake of protein and calium is sufficient, still a high number of older adults with overweight and obesity show insufficient intake. The severity of 25(OH)D deficiency is lowest in older adults with normal weight and highest in older adults with obesity. Healthcare professionals should be made aware of these deficiencies in older adults with overweight and obesity, since there are no guidelines for nutrient deficiencies based on weight.

## Data Availability Statement

The raw data supporting the conclusions of this article will be made available by the authors, without undue reservation.

## Author Contributions

PD: collecting data, the first author of the manuscript, and performed statistical analyses. GR: collecting data, and assisted the first author with all versions of the draft manuscript. CM: collecting data, and assisted the first author with the last draft of the manuscript. WK: performed statistical analyses and assisted in writing the whole manuscript. CS: assisted in writing the complete manuscript. EH and HJ-W: assisted in writing the complete manuscript, assisted in article search/selection when the first author and second author opinion was inconclusive, and assisted in forming the research question and key words for article selection. All authors contributed to the article and approved the submitted version.

## Conflict of Interest

The authors declare that the research was conducted in the absence of any commercial or financial relationships that could be construed as a potential conflict of interest.

## Publisher's Note

All claims expressed in this article are solely those of the authors and do not necessarily represent those of their affiliated organizations, or those of the publisher, the editors and the reviewers. Any product that may be evaluated in this article, or claim that may be made by its manufacturer, is not guaranteed or endorsed by the publisher.

## Abbreviations

25(OH)D: 25-hydroxyvitamin D
BMI: Body mass index
CI: Confidence interval
DRI: Dietary reference intake
ESPEN: European Society for Clinical Nutrition and Metabolism
EFSA: European Food Safety Authority
g/kg: Gram/kilogram body weight
IU: International unit
MeSH: Medical subject headings
MD: Mean difference
N.A.: Not applicable
nmol/L: Nanomole/liter
PRISMA: Preferred Reporting Items for Systematic Reviews and Meta-analyses
RCT: Randomized controlled trial
RE: Random effects
SD: Standard deviation.
