# Supplementary figures and images for: Protein, Calcium, Vitamin D Intake and 25(OH)D Status in Normal Weight, Overweight, and Obese Older Adults: A Systematic Review and Meta-Analysis
Source: Front Nutr. 2021 Sep 10;8:718658. doi: 10.3389/fnut.2021.718658 (PMC8461258; doi:10.3389/fnut.2021.718658)

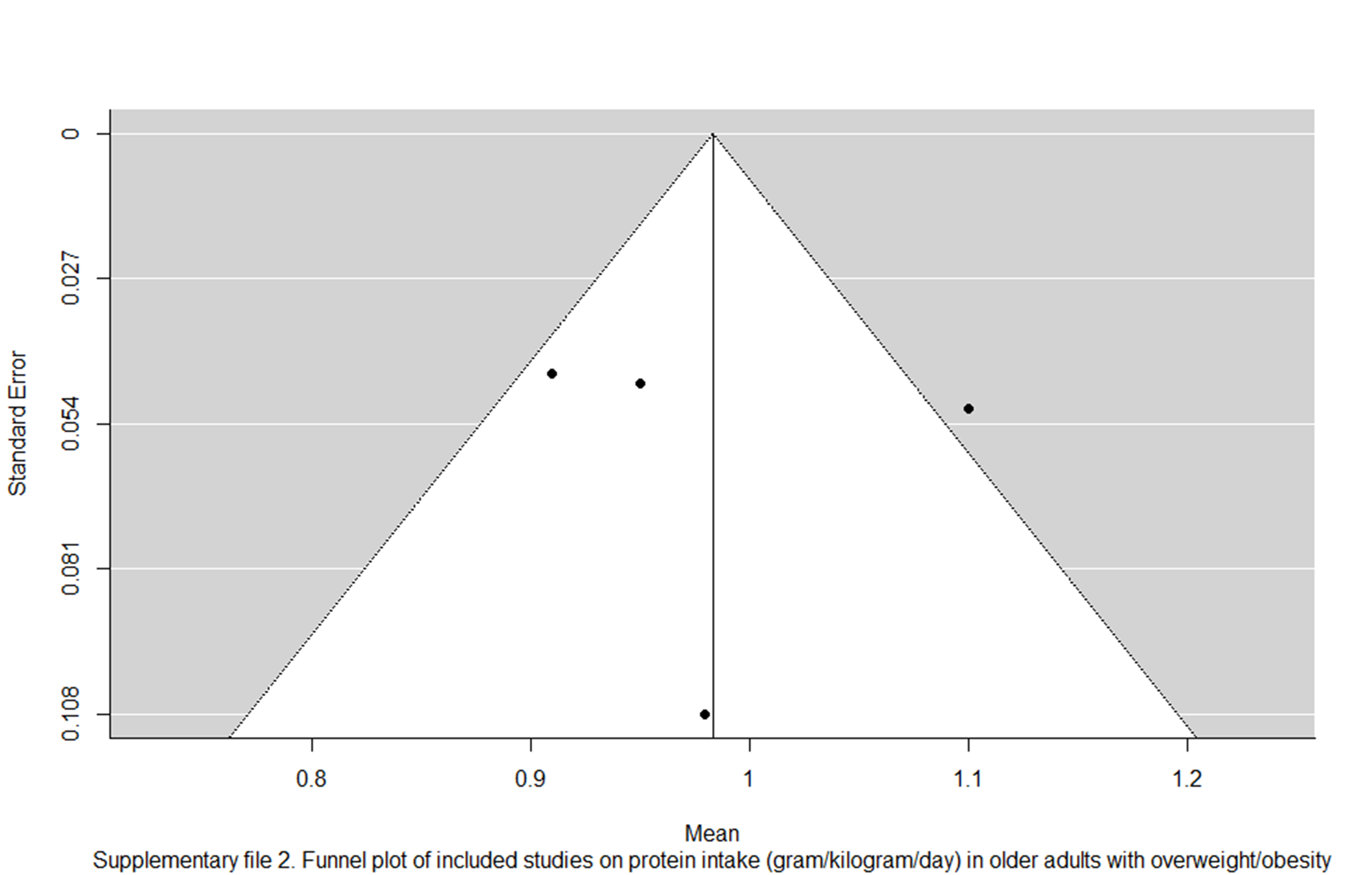

Supplement: Supplementary file 1 [file Image_1.TIFF]

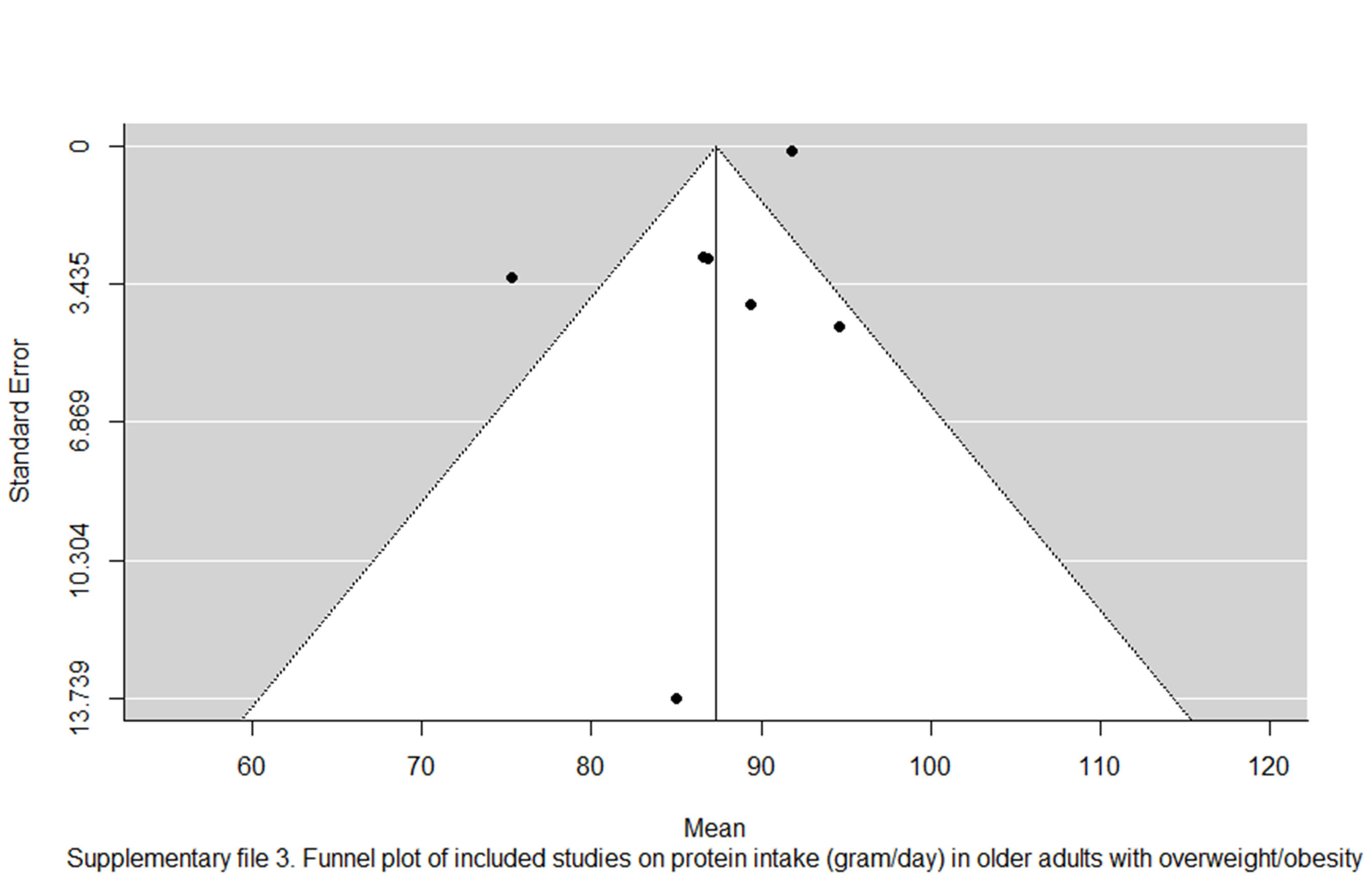

Supplement: Supplementary file 2 [file Image_2.TIFF]

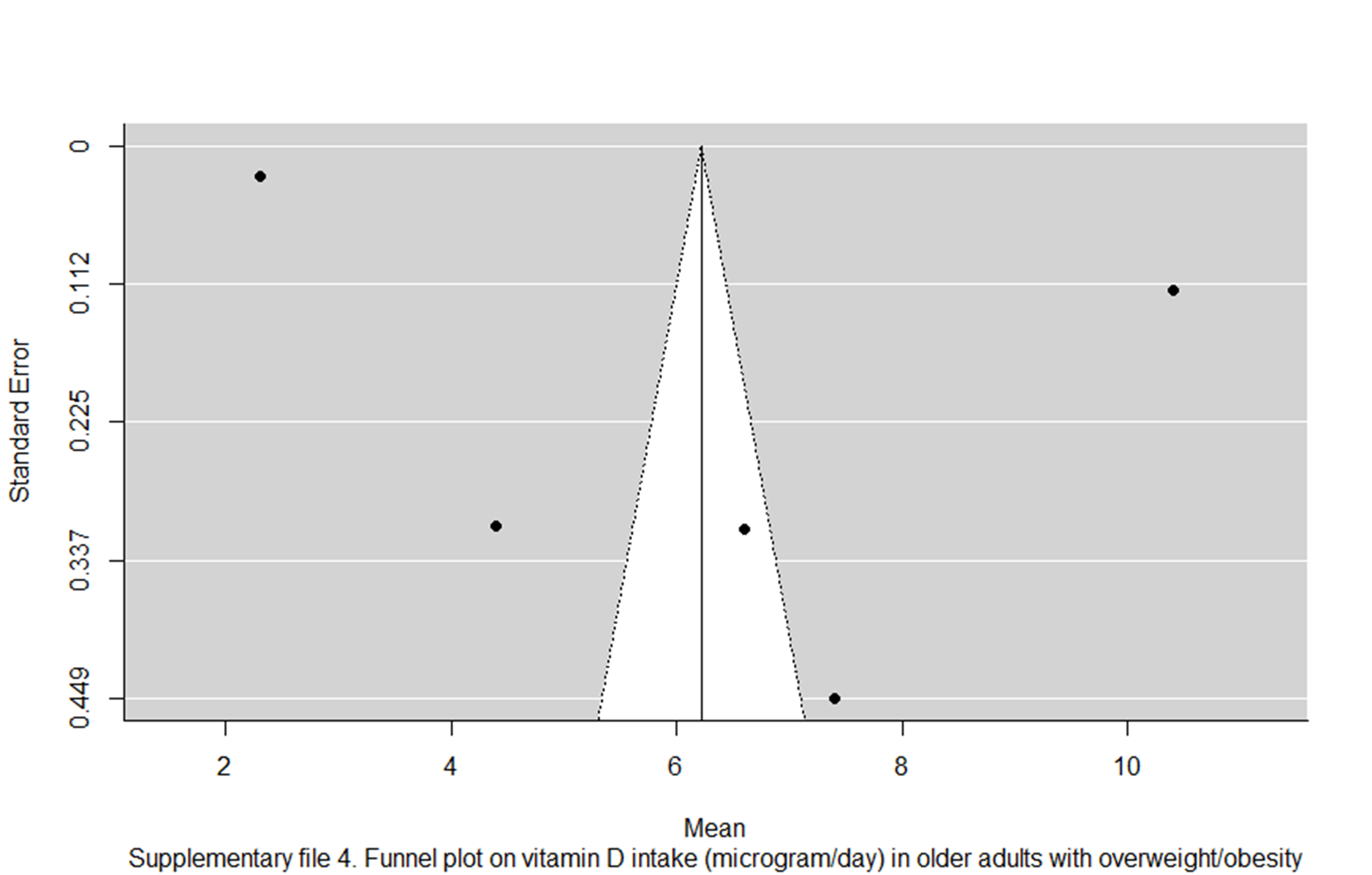

Supplement: Supplementary file 3 [file Image_3.TIFF]

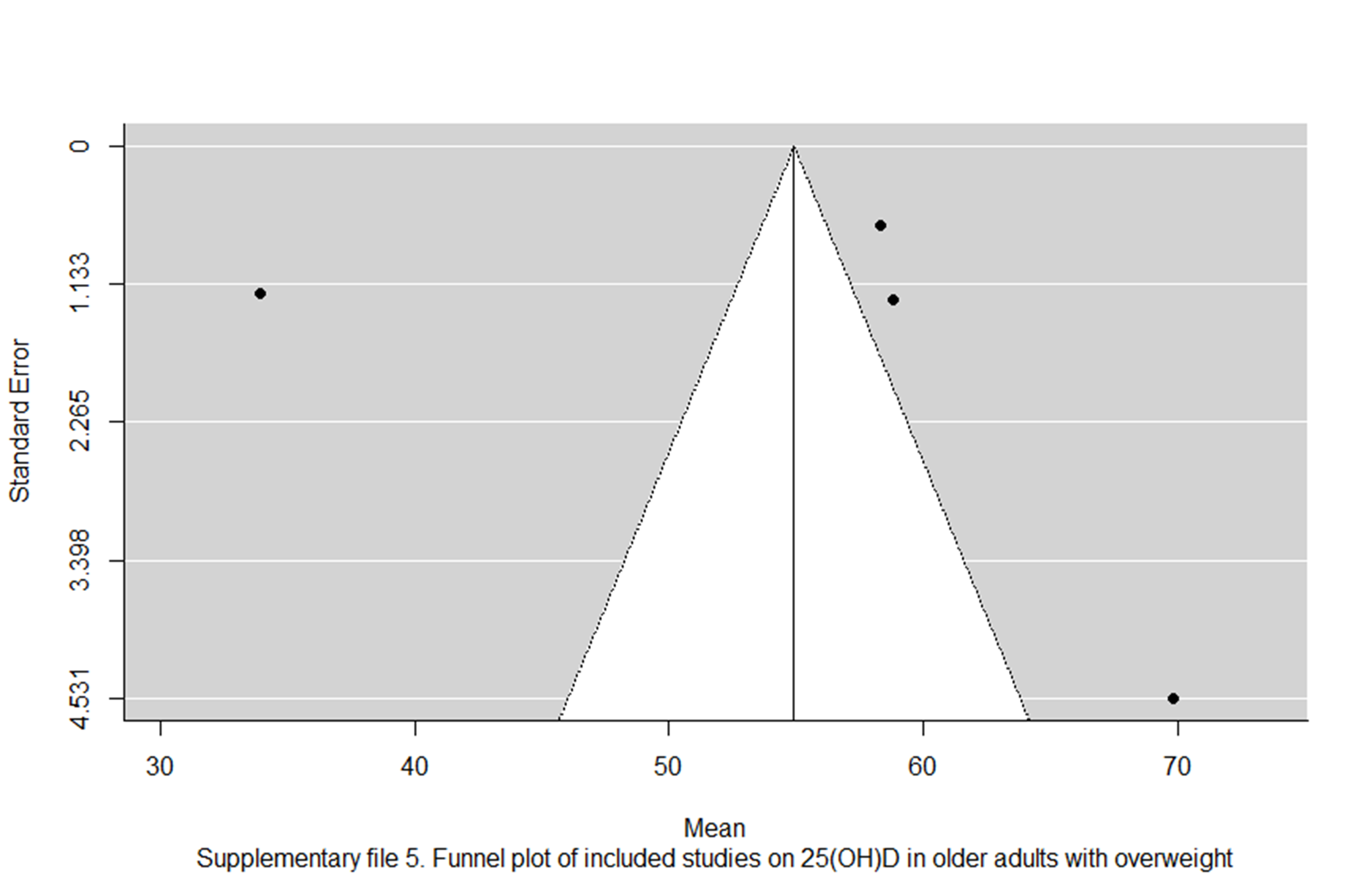

Supplement: Supplementary file 4 [file Image_4.TIFF]

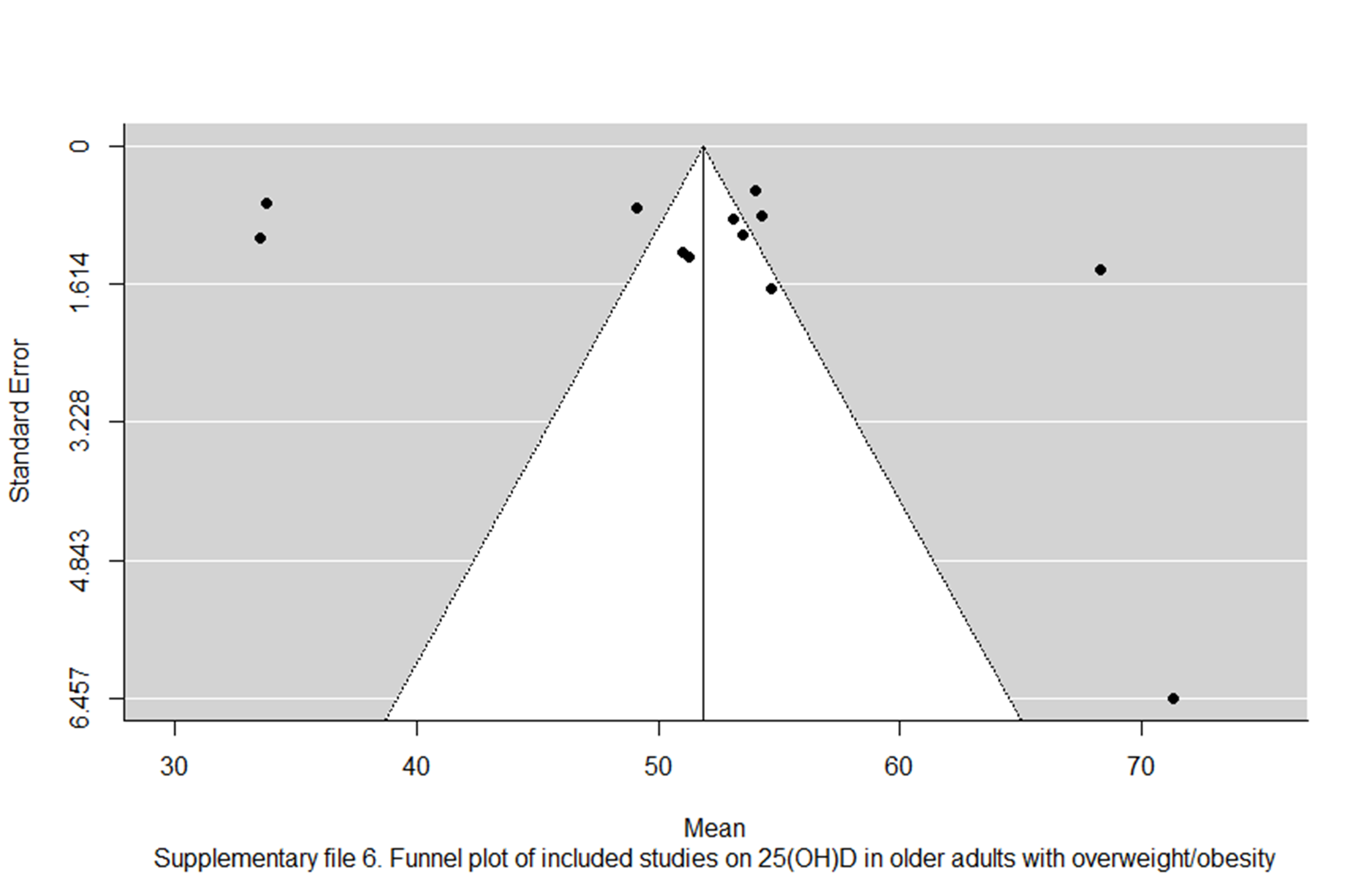

Supplement: Supplementary file 5 [file Image_5.TIFF]

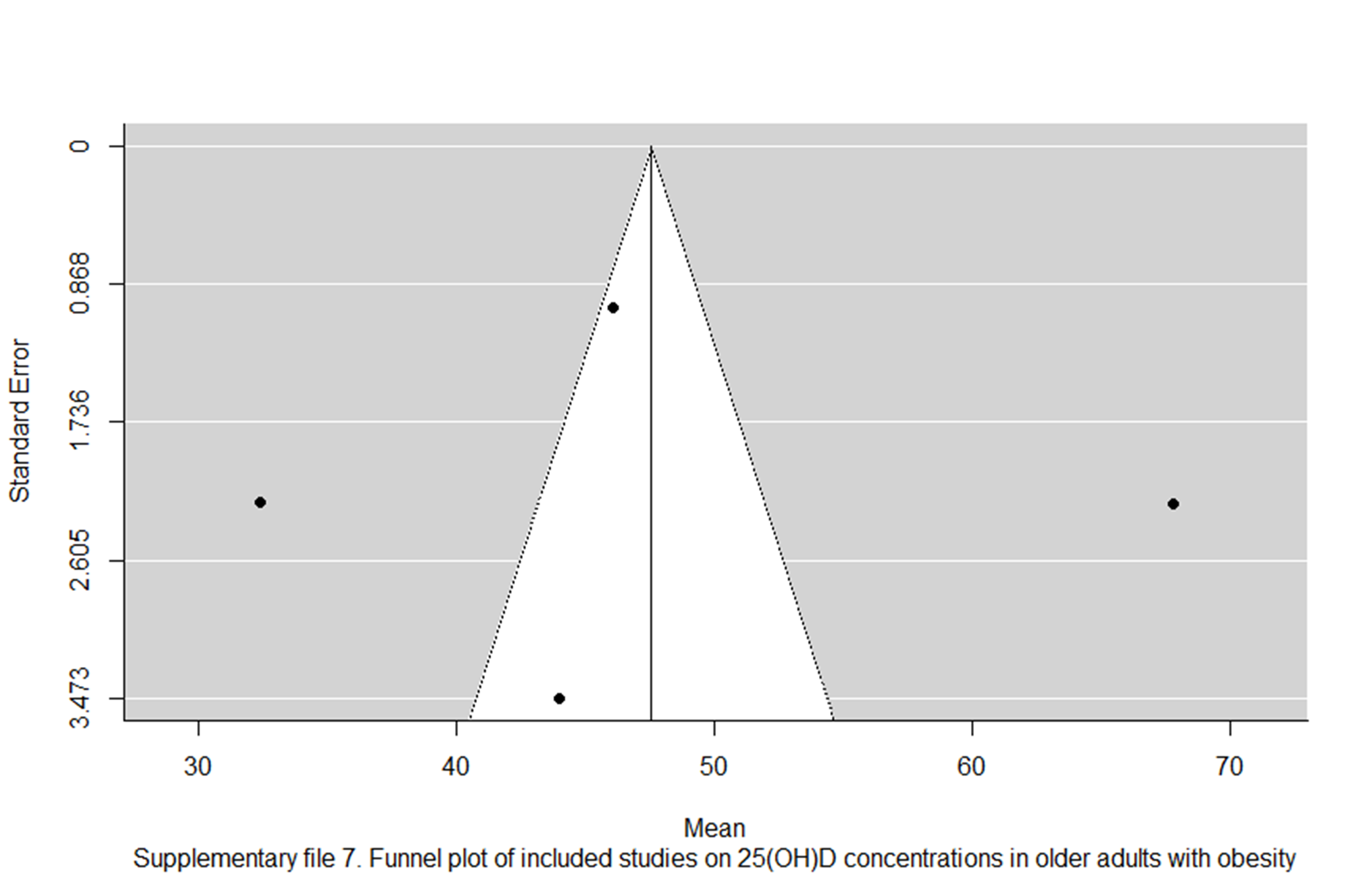

Supplement: Supplementary file 6 [file Image_6.TIFF]

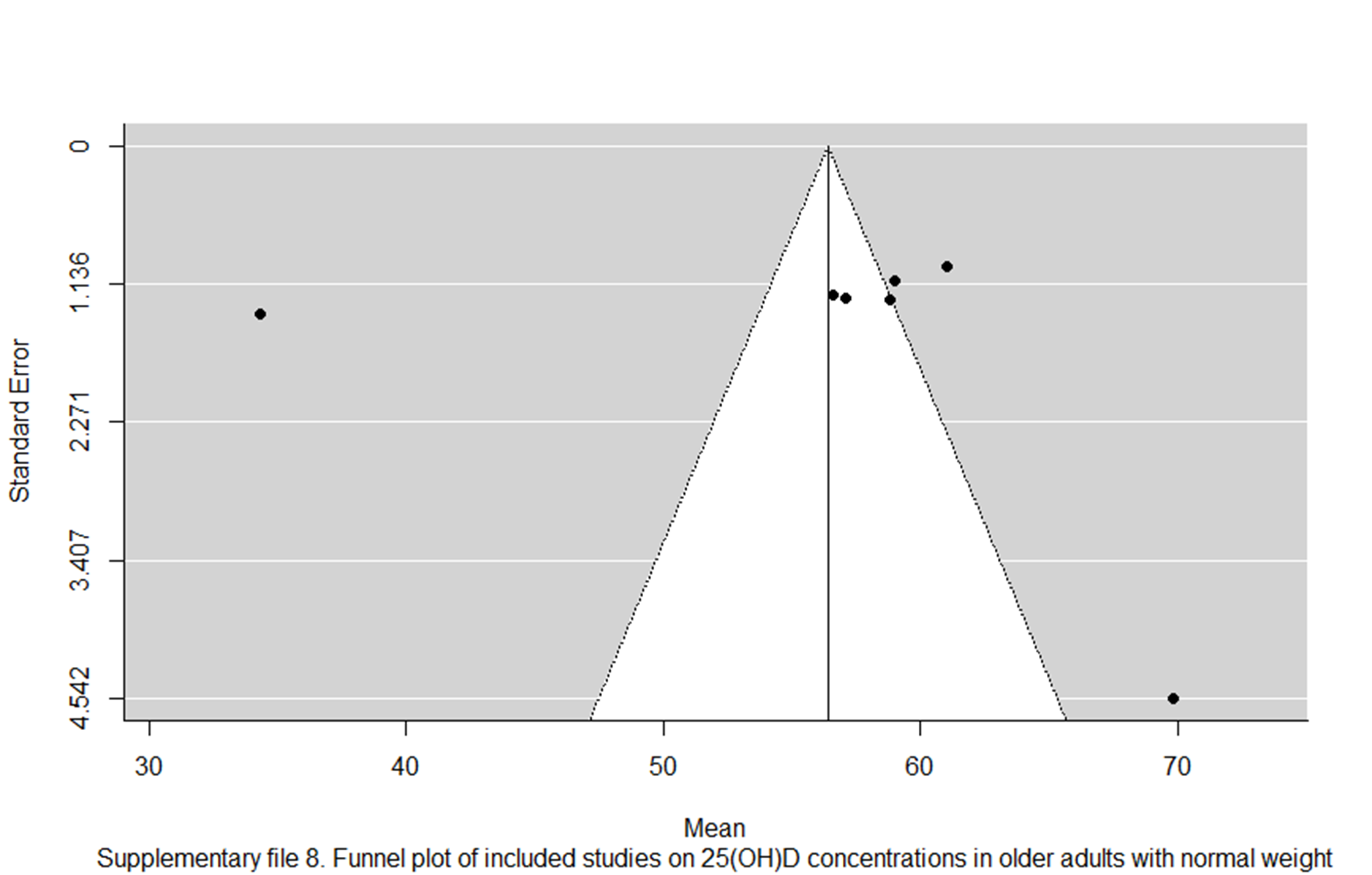

Supplement: Supplementary file 7 [file Image_7.TIFF]

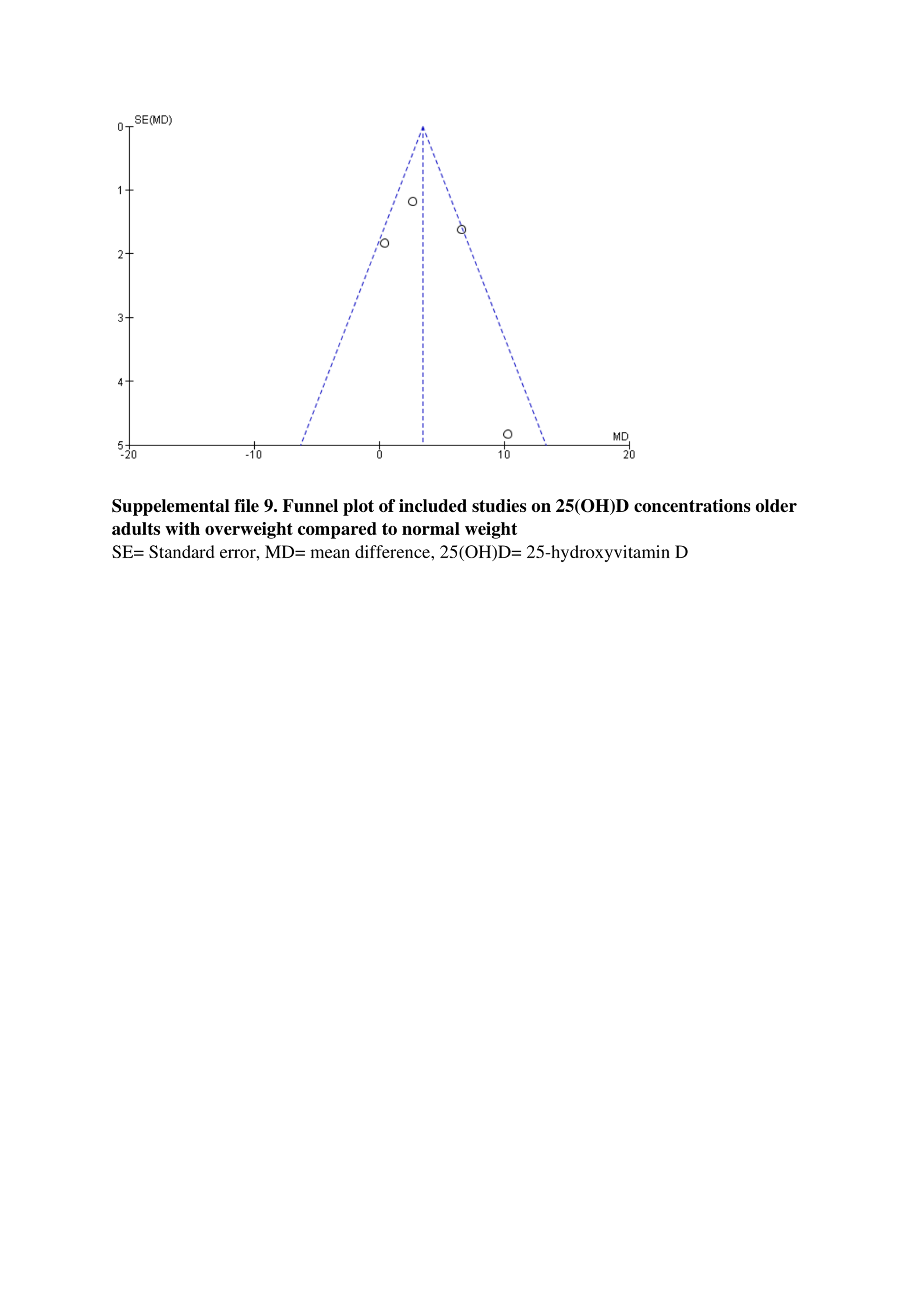

Supplement: Supplementary file 8 [file Image_8.TIFF]

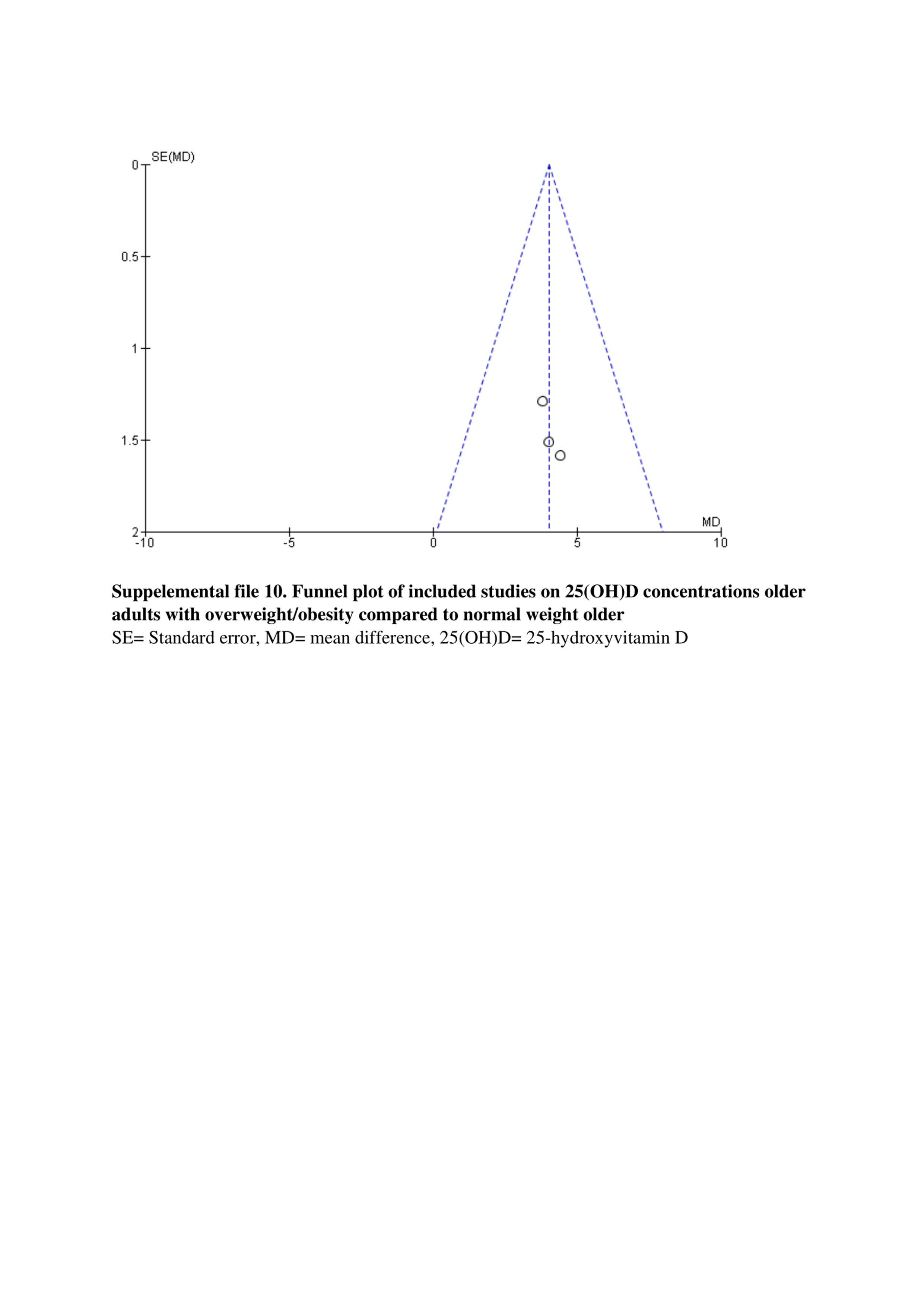

Supplement: Supplementary file 9 [file Image_9.TIFF]

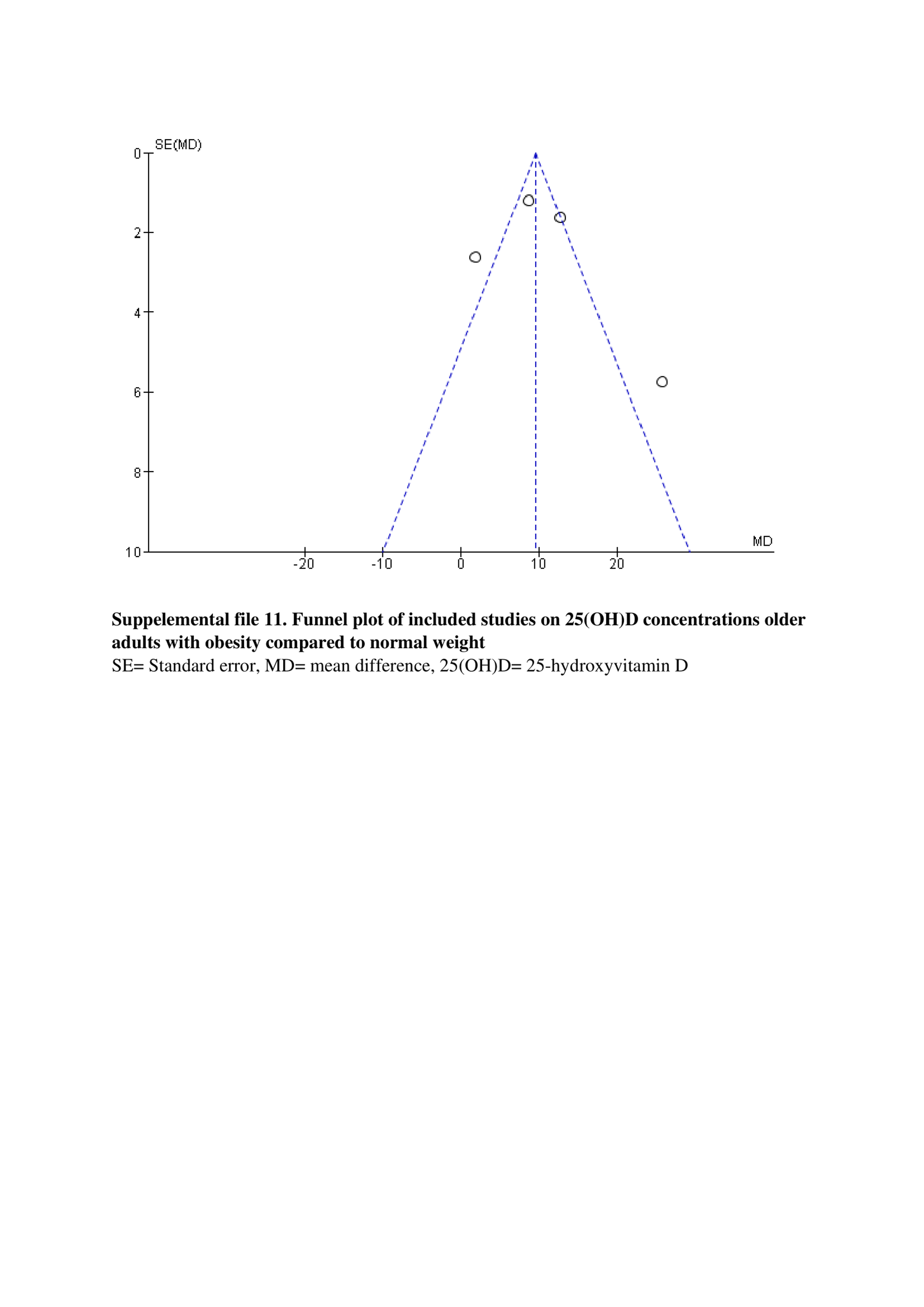

Supplement: Supplementary file 10 [file Image_10.TIFF]

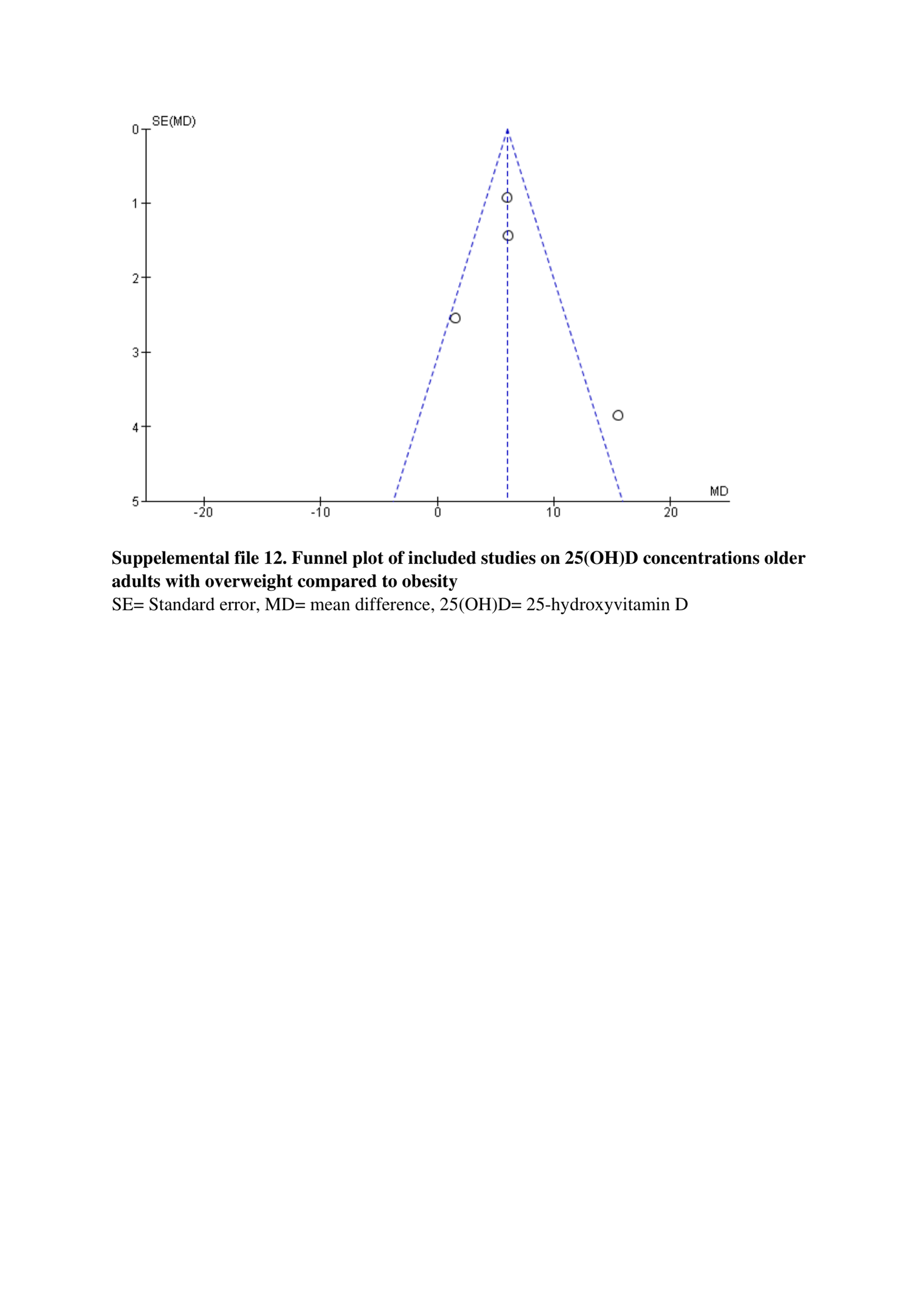

Supplement: Supplementary file 11 [file Image_11.TIFF]

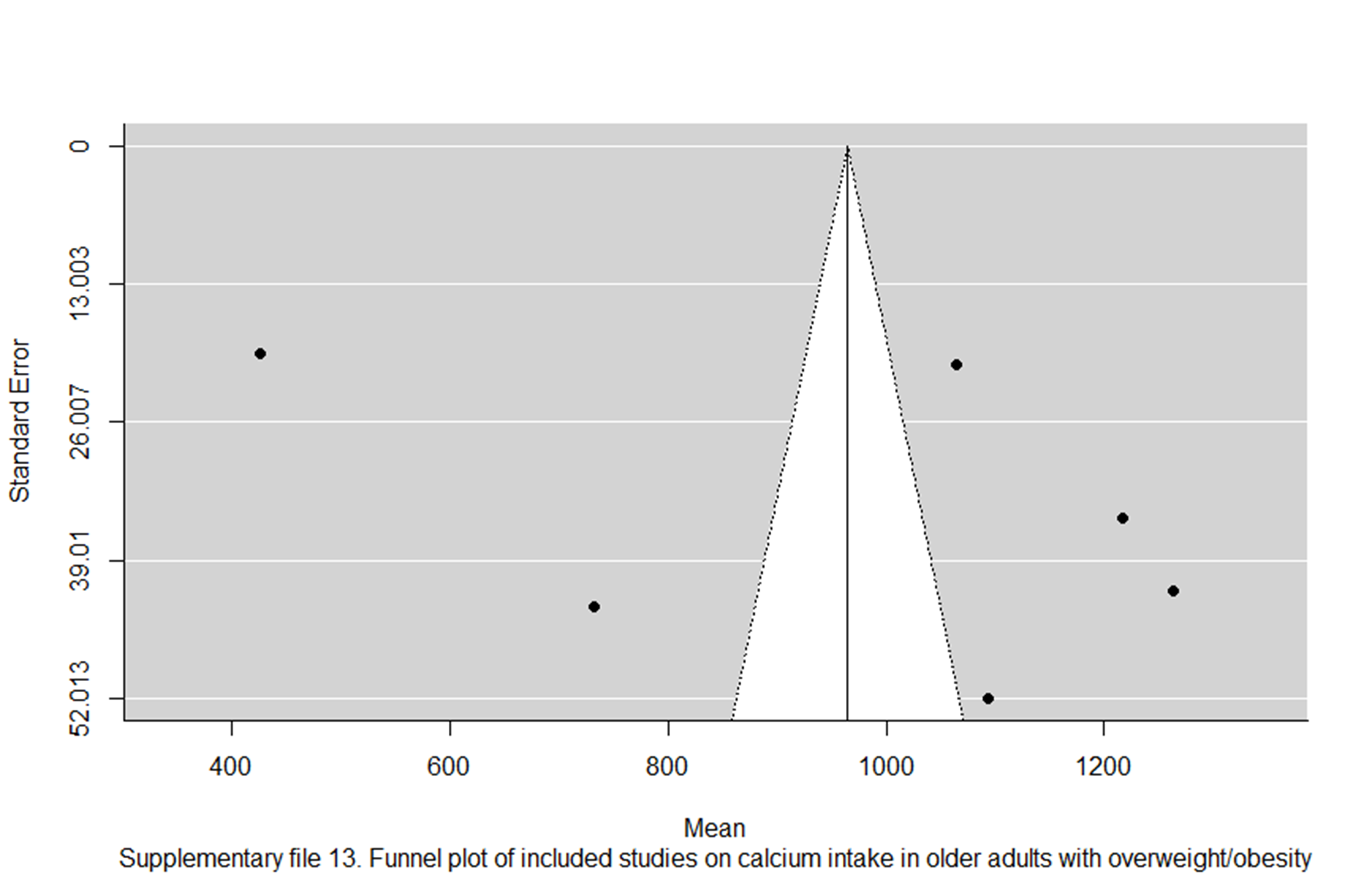

Supplement: Supplementary file 12 [file Image_12.TIFF]

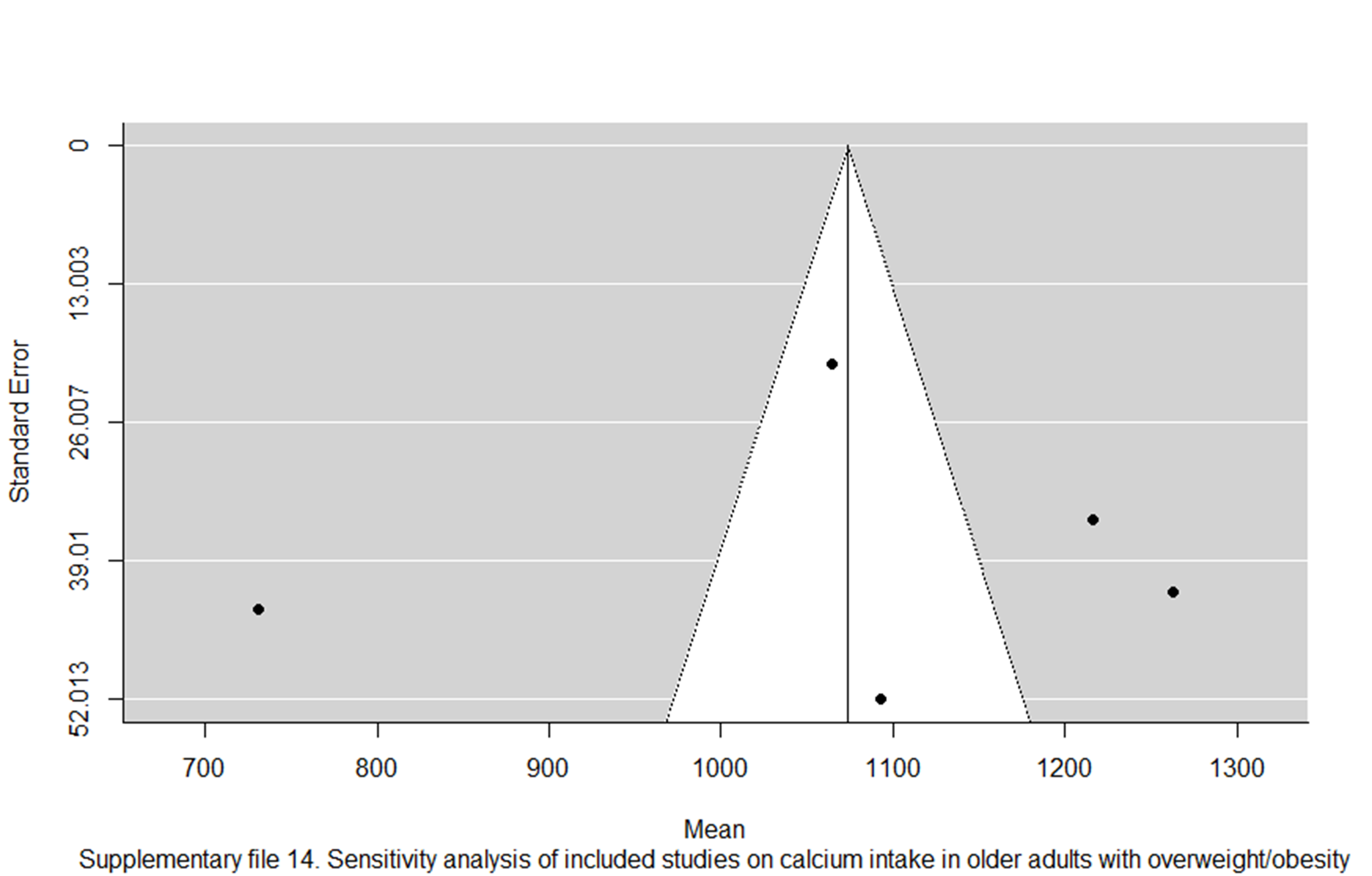

Supplement: Supplementary file 13 [file Image_13.TIFF]
