# Supplementary material for: Protein, Calcium, Vitamin D Intake and 25(OH)D Status in Normal Weight, Overweight, and Obese Older Adults: A Systematic Review and Meta-Analysis
Source: Front Nutr. 2021 Sep 10;8:718658. doi: 10.3389/fnut.2021.718658 (PMC8461258; doi:10.3389/fnut.2021.718658)
Supplement: Supplementary file 14 [file Table_1.DOCX]

# Supplementary file 1. Search strategy for PubMed and Embase **PUBMED**

((((((("aged"[MeSH Terms] OR "middle aged"[MeSH Terms] OR "postmenopause"[MeSH Terms] OR Elder*[tiab] OR Senior*[tiab] OR "Community-dwelling"[tiab] OR Geriatric[tiab] OR "Old people"[tiab] OR "Senior adult"[tiab] OR "Older adult"[tiab] OR "Older adults"[tiab]) AND ((("Overweight"[MeSH Terms] OR "Body Mass Index"[MeSH Terms] OR obese[tiab] OR BMI[tiab] OR obesity[tiab])) AND ((("Vitamin A/administration and dosage"[Mesh] OR "Vitamin A/deficiency"[Mesh] OR "Retinol-Binding Proteins/blood"[Mesh:NoExp] OR (“vitamin A”[tiab] AND intake[tiab]) OR (“vitamin A”[tiab] AND deficien*[tiab]) OR (“vitamin A”[tiab] AND serum[tiab]) OR (“vitamin A”[tiab] and blood[tiab]) OR (retinol[tiab] and blood[tiab]) OR (retinol[tiab] AND serum) OR "Folic Acid/administration and dosage"[Mesh] OR "Folic Acid/deficiency"[Mesh] OR "Homocysteine/blood"[Mesh] OR (“folic acid”[tiab] AND intake[tiab]) OR (“folate”[tiab] AND intake[tiab]) OR (homocysteine[tiab] and serum[tiab]) OR (homocysteine[tiab] and blood[tiab]) OR (“folic acid”[tiab] AND deficien*[tiab]) OR "Vitamin B 12/administration and dosage"[Mesh] OR "Vitamin B 12/deficiency"[Mesh] OR ("vitamin B12"[tiab] AND intake[tiab]) OR ("vitamin B12"[tiab] and serum[tiab]) OR ("vitamin B12"[tiab] and blood[tiab]) OR (“vitamin B12”[tiab] AND deficien*[tiab]) OR "Methylmalonic Acid/blood"[Mesh] OR ("Methylmalonic Acid"[tiab] and serum[tiab]) OR ("Methylmalonic Acid"[tiab] and blood[tiab]) OR ("mma"[tiab] and serum[tiab]) OR ("mma"[tiab] and blood[tiab]) OR "Vitamin D/administration and dosage"[Mesh] OR "Vitamin D/deficiency"[Mesh] OR ("Vitamin D*"[tiab] AND intake[tiab]) OR ("vitamin D*"[tiab] and serum[tiab]) OR ("vitamin D*"[tiab] and blood[tiab]) OR (“vitamin D*”[tiab] AND deficien*[tiab]) OR "Iron, Dietary/administration and dosage"[Mesh] OR "Anemia, Iron-Deficiency/blood"[Mesh] OR "Total iron binding capacity"[tiab] OR "tibc"[tiab] OR (iron[tiab] AND intake[tiab]) OR "Ferritins/blood"[Mesh] OR "Transferrin/blood"[Mesh] OR (ferritin*[tiab] and serum[tiab]) OR (ferritin*[tiab] and blood[tiab]) OR (ferritin*[tiab] and serum[tiab]) OR (ferritin[tiab] and blood[tiab]) OR (transferrin*[tiab] and serum[tiab]) OR (transferrin[tiab] and blood[tiab]) OR (iron[tiab] AND deficien*[tiab]) OR "Calcium/administration and dosage"[Mesh] OR "Bone Density"[Mesh] OR (calcium[tiab] AND intake[tiab]) OR “bone mineral density”[tiab] OR BMD[tiab] OR "Zinc/administration and dosage"[Mesh] OR "Zinc/deficiency"[Mesh] OR (zinc[tiab] AND intake[tiab]) OR (zinc[tiab] and serum[tiab]) OR (zinc[tiab] and blood[tiab]) OR (zinc[tiab] AND deficien*[tiab]) OR "Selenium/administration and dosage"[Mesh] OR "Selenium/deficiency"[Mesh] OR (selenium[tiab] AND intake[tiab]) OR "Selenoproteins/blood"[Mesh] OR "Glutathione Peroxidase/blood"[Mesh] OR (Selenoproteins*[tiab] and serum[tiab]) OR (Selenoproteins*[tiab] and blood[tiab]) OR ("Glutathione Peroxidase”[tiab] and serum[tiab]) OR (“Glutathione peroxidase”[tiab] and blood[tiab]) OR (selenium[tiab] AND deficien*[tiab]) OR "Proteins/administration and dosage"[Mesh] OR "Prealbumin/blood"[Mesh] OR (protein*[tiab] AND intake[tiab]) OR (prealbumin*[tiab] and serum[tiab]) OR (prealbumin*[tiab] and blood[tiab]))) AND ((("Cross-Sectional Studies"[Mesh] OR "Observational Study"[Publication Type] OR "Clinical Study"[Publication Type] OR "Clinical Trial"[Publication Type] OR "Randomized Controlled Trial"[Publication Type] OR "observational study"[tiab] OR "observational studies"[tiab] OR cohort*[tiab] OR "Clinical trial"[tiab] OR "clinical trials"[tiab] OR "cross-sectional study"[tiab] OR "cross-sectional studies"[tiab])) AND (Muscle*[tiab] OR Myofibril[tiab] OR Myofibrils[tiab] OR "Muscles"[Mesh] OR "Muscle, Skeletal"[Mesh] OR "Muscle Strength"[Mesh] OR "Motor Activity"[Mesh] OR “physical function”[tiab] OR “physical functioning”[tiab] OR “Physical performance”[tiab] OR “Physical performing”[tiab] OR “Motor activity”[tiab] OR Exercise*[tiab] OR Mobility[tiab] OR “Functional performance”[tiab] OR “Functional performances”[tiab] OR “Functional test”[tiab] OR “Functional tests”[tiab] OR "Quality of Life"[Mesh] OR "Quality-Adjusted Life Years"[Mesh] OR “QoL”[tiab] OR "Life quality"[tiab] OR "Life qualities"[tiab] OR "Disability-Adjusted Life Year"[tiab] OR “DALY”[tiab] OR “QUALY”[tiab] OR "Quality-adjusted life year"[tiab] OR "Bone and Bones"[Mesh] OR "Fractures, Bone"[Mesh] OR "Bone Density"[Mesh] OR "Osteoporosis, Postmenopausal"[Mesh] OR "Osteoporosis"[Mesh] OR "procollagen Type I N-terminal peptide"[Supplementary Concept] OR "procollagen type I carboxy terminal peptide"[Supplementary Concept] OR "tartrate-resistant acid phosphatase"[Supplementary Concept] OR Bone[tiab] OR Fracture*[tiab] OR “bone specific alkaline phosphatase”[tiab] OR osteopenia[tiab] OR Osteoporosis[tiab] OR Skelet*[tiab] OR “Procollagen type I N-propeptide*”[tiab] OR P1NP[tiab] OR “Procollagen type I C propeptide*”[tiab] OR P1CP*[tiab] OR “Deoxypyridinoline N-telopeptides”[tiab] OR NTx*[tiab] OR “C-terminal”[tiab] OR “Telopeptides of type I collagen*”[tiab] OR CTX*[tiab] OR “Tartrate-resistant acid”[tiab] OR TRACP[tiab] OR TRAP 5b*[tiab]) AND "last 10 years"[PDat] AND Humans[Mesh]) NOT child* NOT infant* NOT renal NOT kidney NOT "Child"[Mesh] NOT birth* NOT "Kidney"[Mesh]))

**EMBASE**

**click off: Search as broadly as possible (map, explode, search also as free text in all fields))**

(human OR 'human'/exp) AND ('aged'/exp OR 'aged':ab,ti OR 'postmenopause'/exp OR ageing:ab,ti OR aging:ab,ti OR elder:ab,ti OR senior*:ab,ti OR 'community dwelling':ab,ti OR geriatric:ab,ti) AND ('obesity'/exp OR obese:ab,ti OR obesity:ab,ti OR bmi:ab,ti OR 'body mass index':ab,ti OR overweight:ab,ti) AND (retinol:ab,ti AND intake:ab,ti OR ('vitamin a':ab,ti AND deficiency:ab,ti) OR (retinol:ab,ti AND deficiency:ab,ti) OR ('vitamin a':ab,ti AND serum:ab,ti) OR ('vitamin a':ab,ti AND blood:ab,ti) OR ('vitamin a':ab,ti AND plasma:ab,ti) OR (retinol:ab,ti AND serum:ab,ti) OR (retinol:ab,ti AND blood:ab,ti) OR (retinol:ab,ti AND plasma:ab,ti) OR ('folic acid':ab,ti AND intake:ab,ti) OR (folate:ab,ti AND intake:ab,ti) OR 'folic acid deficiency'/exp OR ('folic acid':ab,ti AND deficiency:ab,ti) OR (folate:ab,ti AND deficiency:ab,ti) OR ('folic acid':ab,ti AND serum:ab,ti) OR ('folic acid':ab,ti AND blood:ab,ti) OR ('folic acid':ab,ti AND plasma:ab,ti) OR (folate:ab,ti AND serum:ab,ti) OR (folate:ab,ti AND blood:ab,ti) OR (folate:ab,ti AND plasma:ab,ti) OR 'homocysteine'/exp OR ('homocysteine':ab,ti AND blood:ab,ti) OR ('homocysteine':ab,ti AND serum:ab,ti) OR ('homocysteine':ab,ti AND plasma:ab,ti) OR ('vitamin b12':ab,ti AND intake:ab,ti) OR 'vitamin b12 deficiency'/exp OR ('vitamin b12':ab,ti AND deficiency:ab,ti) OR ('vitamin b12':ab,ti AND serum:ab,ti) OR ('vitamin b12':ab,ti AND blood:ab,ti) OR ('vitamin b12':ab,ti AND plasma:ab,ti) OR ('methylmalonic acid':ab,ti AND serum:ab,ti) OR ('methylmalonic acid':ab,ti AND blood:ab,ti) OR ('methylmalonic acid':ab,ti AND plasma:ab,ti) OR ('mma':ab,ti AND serum:ab,ti) OR ('mma':ab,ti AND blood:ab,ti) OR ('mma':ab,ti AND plasma:ab,ti) OR ('vitamin d':ab,ti AND intake:ab,ti) OR 'vitamin d deficiency'/exp OR ('vitamin d':ab,ti AND serum:ab,ti) OR ('vitamin d':ab,ti AND blood:ab,ti) OR ('vitamin d':ab,ti AND plasma:ab,ti) OR (iron:ab,ti AND intake:ab,ti) OR ('ferritin':ab,ti AND serum:ab,ti) OR ('ferritin':ab,ti AND blood:ab,ti) OR ('ferritin':ab,ti AND plasma:ab,ti) OR ('transferrin':ab,ti AND serum:ab,ti) OR ('transferrin':ab,ti AND blood:ab,ti) OR ('transferrin':ab,ti AND plasma:ab,ti) OR 'iron deficiency'/exp OR (iron:ab,ti AND deficiency) OR 'calcium intake'/exp OR (calcium:ab,ti AND intake:ab,ti) OR ('calcium':ab,ti AND serum:ab,ti) OR ('calcium':ab,ti AND blood:ab,ti) OR ('calcium':ab,ti AND plasma:ab,ti) OR 'bone density'/exp OR 'bone mineral density':ab,ti OR bmd:ab,ti OR ('zinc':ab,ti AND intake:ab,ti) OR 'zinc deficiency'/exp OR (zinc:ab,ti AND deficiency:ab,ti) OR ('zinc':ab,ti AND serum:ab,ti) OR ('zinc':ab,ti AND blood:ab,ti) OR ('zinc':ab,ti AND plasma:ab,ti) OR (selenium:ab,ti AND intake:ab,ti) OR 'selenium deficiency'/exp OR (selenium:ab,ti AND deficiency) OR ('selenium':ab,ti AND serum:ab,ti) OR ('selenium':ab,ti AND blood:ab,ti) OR ('selenium':ab,ti AND plasma:ab,ti) OR (selenoprotein:ab,ti AND blood:ab,ti) OR (selenoprotein:ab,ti AND serum:ab,ti) OR (selenoprotein:ab,ti AND plasma:ab,ti) OR ('glutathione peroxidase':ab,ti AND blood:ab,ti) OR ('glutathione peroxidase':ab,ti AND serum:ab,ti) OR ('glutathione peroxidase':ab,ti AND plasma:ab,ti) OR 'protein intake'/exp OR (protein*:ab,ti AND intake:ab,ti) OR 'prealbumin'/exp OR ('prealbumin':ab,ti AND blood:ab,ti)) AND ('clinical trial'/exp OR 'clinical trial':ab,ti OR 'prospective studies'/exp OR 'prospective studies':ab,ti OR 'intervention study'/exp OR 'intervention study':ab,ti OR 'longitudinal study'/exp OR 'longitudinal study':ab,ti OR 'major clinical study'/exp OR 'major clinical study':ab,ti OR 'experimental study'/exp OR 'experimental study':ab,ti OR 'randomized controlled trial'/exp OR 'randomized controlled trial':ab,ti) AND [humans]/lim AND ([middle aged]/lim OR [aged]/lim OR [very elderly]/lim) NOT ([embryo]/lim OR [fetus]/lim OR [newborn]/lim OR [infant]/lim OR [child]/lim OR [preschool]/lim OR [school]/lim OR [adolescent]/lim OR [young adult]/lim)
